# Direct myosin-2 inhibition enhances cerebral perfusion resulting in functional improvement after ischemic stroke

**DOI:** 10.7150/thno.42077

**Published:** 2020-04-06

**Authors:** Máté Pénzes, Demeter Túrós, Domokos Máthé, Krisztián Szigeti, Nikolett Hegedűs, Anna Ágnes Rauscher, Péter Tóth, Ivan Ivic, Parasuraman Padmanabhan, Gabriella Pál, Árpád Dobolyi, Máté Gyimesi, András Málnási-Csizmadia

**Affiliations:** 1 MTA-ELTE Motor Pharmacology Research Group, Eötvös Loránd University, Department of Biochemistry, Pázmány Péter sétány 1/c, 1117 Budapest, Hungary; 2 Department of Biophysics and Radiation Biology, Nanobiotechnology and In Vivo Imaging Centre, Semmelweis University, Tűzoltó utca 37-47, 1094 Budapest, Hungary; 3 Department of Neurosurgery, University of Pécs, Medical School, Szigeti út 12, 7624 Pécs, Hungary; 4 MTA-PTE Clinical Neuroscience MR Research Group-Pécs Diagnostic Center, Pécs, Hungary; 5 Cognitive Neuroimaging Centre (CoNiC), Lee Kong Chian School of Medicine, 59, Nanyang Drive, Nanyang Technological University, Singapore-636921; 6 MTA-ELTE Laboratory of Molecular and Systems Neurobiology, Department of Physiology and Neurobiology, Eötvös Loránd University and the Hungarian Academy of Sciences, Pázmány Péter sétány 1/c, 1117 Budapest, Hungary; 7 Hungarian Defense Forces Military Hospital, Róbert Károly körút 44, 1134 Budapest, Hungary

**Keywords:** MCAO, reperfusion, myosin, smooth muscle cell, SPECT

## Abstract

Acute ischemic stroke treatment faces an unresolved obstacle as capillary reperfusion remains insufficient after thrombolysis and thrombectomy causing neuronal damage and poor prognosis. Hypoxia-induced capillary constriction is mediated by actomyosin contraction in precapillary smooth muscle cells (SMCs) therefore smooth muscle myosin-2 could be an ideal target with potentially high impact on reperfusion of capillaries.

**Methods**: The myosin-2 inhibitor *para*-aminoblebbistatin (AmBleb) was tested on isolated human and rat arterioles to assess the effect of AmBleb on vasodilatation. Transient middle cerebral artery occlusion (MCAO) was performed on 38 male Wistar rats followed by local administration of AmBleb into the ischemic brain area. Development of brain edema and changes in cerebrovascular blood flow were assessed using MRI and SPECT. We also tested the neurological deficit scores and locomotor asymmetry of the animals for 3 weeks after the MCAO operation.

**Results**: Our results demonstrate that AmBleb could achieve full relaxation of isolated cerebral arterioles. In living animals AmBleb recovered cerebral blood flow in 32 out of the 65 affected functional brain areas in MCAO operated rats, whereas only 8 out of the 67 affected areas were recovered in the control animals. Animals treated with AmBleb also showed significantly improved general and focal deficit scores in neurological functional tests and showed significantly ameliorated locomotor asymmetry.

**Conclusion**: Direct inhibition of smooth muscle myosin by AmBleb in pre-capillary SMCs significantly contribute to the improvement of cerebral blood reperfusion and brain functions suggesting that smooth muscle myosin inhibition may have promising potential in stroke therapies as a follow-up treatment of physical or chemical removal of the occluding thrombus.

## Introduction

Acute ischemic stroke is an ever-increasing disease affecting the entire world's population despite the wide range of efforts to improve preventive strategies 1. Ischemic stroke treatment with special focus on regeneration is of immediate and serious medical and economic interest due to the high occurrence of stroke and its serious lifelong consequences 2. Stroke treatment with intra-arterial thrombolytics, such as plasminogen activators still remain the mainstay intervention *per se* or in combination with mechanical thrombectomy 3, which must be further evolved to decrease the mortality rate of the disease and to ameliorate the consequences for the stroke survivors 2. These treatments should involve both acute and prolonged interventions including enhanced add-on therapies for mechanical thrombectomy. The major unresolved issue in stroke treatment is that cerebral capillaries remain closed by hypoxia-induced contraction of precapillary smooth muscle cells (SMCs) 4, 5 even after opening the large vessels by thrombectomy. The permanently constricted small vessels dramatically suppress tissue blood supply contributing to ultimate cell death in the penumbra. Therefore, a high unmet need is in the field for drug treatments enhancing restoration of local oxygen supply in the penumbra following hypoxia-induced ischemic cascade 6, 7. Directly targeting the contractile apparatus of precapillary SMCs with a small molecule inhibitor may fulfill both short- and long-term requirements towards an innovative medication. In addition, a smooth muscle myosin (SMM) targeting treatment combined with thrombectomy and/or thrombolysis has the potential to extend the treatment-eligibility criteria thereby allowing effective therapies to a broader population of stroke patients.

Restriction of blood supply during ischemic stroke was demonstrated to trigger the constriction of smooth muscle actin expressing contractile SMCs followed by SMC death in rigor 4, 5. SMC contraction is most presumably due to complex cellular processes: a few minutes after ischemic stroke anoxic depolarization of cells induces the rise of intracellular Ca^2+^ release facilitating smooth muscle contraction. Moreover, hypoxia is followed by the decline in ATP levels, which hinders the detachment of myosin from actin thereby populating myosin in the strongly actin-bound, rigor state. In addition, the formation of oxygen and nitrogen radicals during ischemia also contributes to pericyte constriction 4, 8, 9.

These events lead to the irreversible contraction of brain capillaries resulting in prolonged decrease in blood flow, downstream thrombus formation and the concomitant damages of neurons.

The most promising advances in regeneration after stroke have been achieved by influencing the Rho-associated kinase (ROCK) pathway. ROCK is a central hub of several different pathways regulating protein synthesis, cell growth, cytoskeleton rearrangements and acto-myosin contraction in non-muscle cell cortices 10, 11. Importantly, ROCK pathway is responsible for smooth muscle contraction in SMC-s through SMM activation thereby regulating blood pressure 12, 13. Furthermore, intravenous administration of liposomal ROCK-inhibitor fasudil 14 was effective in recovery after stroke in rodent transient middle cerebral artery occlusion (MCAO) stroke models. However, the improvement of blood flow after ischemic stroke was not demonstrated in fasudil treated animals.

We hypothesized that direct inhibition of SMM in pre-capillary SMCs might achieve the same positive effects as ROCK inhibition while avoiding the unwanted negative side effects of inhibiting an upstream hub regulator protein. In this study, we demonstrate that direct inhibition of SMM with a biologically safe blebbistatin derivative *para*-aminoblebbistatin (AmBleb) 15 restores cerebral blood perfusion and significantly improves neurological functions in MCAO operated rat stroke model.

## Materials and Methods

### Materials

Chemicals were purchased from Sigma-Aldrich (Germany) if otherwise not stated. [^99m^Tc]-HMPAO Brain-SPECT® kit was purchased from Medi-Radiopharma Co., Ltd. (Hungary). StemPro^™^ Neural Stem Cells and the required chemicals for cells were purchased from Thermo Fisher Scientific (USA). The isoflurane was purchased from Medicus Partner Ltd. (Hungary).

### Purification of actin and smooth muscle myosin 2

Rabbit actin was prepared as described previously 16. Smooth muscle myosin was prepared from chicken gizzard 17 and subfragment-1 (labeled as SMM in the manuscript for simplicity because we do not use other smooth muscle myosin forms) was prepared by activated papain digestion 18 (0.2 mg/ml papain, 12 min, 25^o^C, reaction was stopped with 5 mM sodium iodoacetate). SMM concentration was determined with Bradford Protein Assay.

### ATPase activity measurements

Steady-state ATPase measurements were carried out in 50 μl in a flat bottom 384-well plate (Nunc-Thermo Fischer) using NADH-PK/LDH coupled assay described previously 19 at 25^o^C in the presence of 0.5 mM ATP and F-actin (25 μM for NM2s and 33 μM for SmS1) in ATPase buffer (10 mM MOPS pH 7.0, 4 mM MgCl_2_, 2 mM β-mercaptoethanol) for 15 minutes. Three parallel measurements were carried out for each point. Different concentration of AmBleb was added to the reaction in 0.5 μl DMSO (1% of total volume). DMSO and actin-controls were measured to each measurement set. ATPase activity was calculated from linear regression of the time dependent absorbance data collected at 340 nm.

### Vessel contraction assay - Vasomotor responses of rat and human cerebral parenchymal arterioles to AmBleb

Following written informed consent and approved by the Regional Ethic and Review Committee of the University of Pécs (3887), we obtained cortical samples from patients undergoing neurosurgical removal of cerebral tumors without known comorbidities, which would have been discarded, as published previously 20. In brief, preoperatively contrast enhanced magnetization prepared rapid acquisition gradient echo (MP-RAGE) MRI sequence was performed to visualize contrast enhancing areas with pathologically increased blood brain barrier permeability. Cerebral samples and/or the arterioles themselves were removed carefully from non-enhancing normal cortical areas that had to be removed to approach deep-seated tumors 20. Cerebral tissue from the fronto-temporo-parietal lobes was placed in 0°C-4°C physiologic salt solution (PSS, 110.0 NaCl, 5.0 KCl, 2.5 CaCl_2_, 1.0 MgSO_4_, 1.0 KH_2_PO_4_, 5.5 glucose, and 24.0 mM NaHCO_3_ equilibrated with a gas mixture of 95% air and 5% CO_2_, pH ∼7.3). Afterward, intraparenchymal arterioles were isolated under an operating microscope, cut into rings and transferred into a wire myograph (Danish Myo Technology 610M, Aarhus, Denmark). Arterioles' segments (1.5-2 mm in length) were suspended between two 40 µm tungsten wires in the myograph chambers containing constantly oxygenated PSS (36.9 ± 0.1°C). In separate series of experiments, approved by the Institutional Animal Use and Care Committee of the University of Pecs Medical School and the National Scientific Ethical Committee on Animal Experimentation, Hungary (BAI/35/51-107/2016) rats (n=6) were anesthetized, decapitated and basilar arteries (BA) were isolated and studied in the wire myograph. Before the start of an experiment, length-tension curve was obtained to determine the optimal passive tension for each vessel, then vessels were allowed to for one hour to equilibrate, according to the manufacturer's instructions. Endothelial function was tested by relaxation responses to adenosine-triphosphate (ATP; from 10^-9^ to 10^-4^ M, Sigma Aldrich). Endothelium-independent relaxation was studied by administration of the NO donor sodium nitroprusside (from 10^-9^ to 10^-4^ M, Sigma Aldrich). Contractile ability of the vessels was tested by obtaining vasomotor responses to the beta-adrenergic agonist norepinephrine (from 10^-9^ to 10^-5^ M, Sigma Aldrich). After the vessels reached maximal contraction (plateau phase) in response to 60 mM KCl, vasomotor responses were assessed to cumulative concentrations of AmBleb (1, 10 and 30 µM for 15 min each). After AmBleb treatment, vasoconstrictor and vasorelaxation functional reactivity were re-assessed.

### Animals

Experiments were conducted on male Wistar rats (200-250 g body weight), bred in a Specific Pathogen Free unit at Toxi-Coop Ltd. (Budapest, Hungary). Animals were maintained in standard housing conditions with 12-hour light, 12-hour dark periods and were allowed free access to dry rat food and water. All procedures were conducted in accordance with the ARRIVE guidelines and the guidelines set by the European Communities Council Directive (86/609 EEC) and approved by the Animal Care and Use Committee of the Semmelweis University (protocol number: XIV-I-001/29-7/2012). Three animals were operated daily, animals were assigned to control or treated groups randomly.

*In vivo* studies started only after we confirmed the effectiveness of AmBleb in numerous *in vitro* and cellular assays. We also aimed to first demonstrate the *in vivo* effectiveness of AmBleb on rodent animal models before recruiting larger animals, however, anatomical differences between the rodent and human brain vasculature may pose limitation to the translation of the applied methodology to human subjects. Furthermore, we made efforts to reduce the number of animals involved in the study by applying advanced biomedical imaging methods combined with the development of unbiased analysis methods. We kept animal numbers at a minimum level with which statistically significant results could be achieved.

### Middle Cerebral Artery Occlusion

An intraluminal invasive endovascular surgical procedure, the Koizumi-type transient middle cerebral artery occlusion (MCAO) 21 was performed to model ischemic stroke by occluding the middle cerebral artery (MCA) in rats. Wistar rats and silicone filament coating were used to minimize mortality and variability 22. Briefly, animals were pre-anesthetized with 4% isoflurane in medical oxygen in a closed plastic induction box. Once pre-anesthesia had taken place, the animal was transferred to the surgery table and fitted into a nose cone and isoflurane was reduced to 2% for maintenance. Firstly, a midline neck incision was carried out and the left common carotid artery (CCA), external carotid artery (ECA) and internal carotid artery (ICA) were exposed precisely and isolated from the surrounding tissues. After ligation of the CCA and ECA with silk suture (Silk suture USP 1, KRUUSE, Langeskov, Denmark), a 4-0 silicone rubber-coated monofilament (4-0 Medium B MCAO suture L45 PK10, Doccol Corporation, Sharon, MA, USA) was inserted through the CCA into the ICA 20 mm beyond the carotid bifurcation to occlude the MCA for 1 hour.

### Delivery of AmBleb into the brain

The 1 hour-long MCAO was followed by the injection of either AmBleb (patent application number: EP16153446, 25 mM dissolved in DMSO) or vehicle into the left damaged hemisphere of the brain. Firstly, an anterior-posterior incision between the ears was carried out by a scalpel and kept open with surgery clamps. The surface of the skull was cleaned with H_2_O_2_ until the intersection between the coronal suture and sagittal sutures (bregma point) were visible. The injection coordinates were measured from the bregma using coordinates (AP +0.3 mm, ML -4.0 mm, DV +4.8 mm) according to a rat brain atlas 23. A 10 µl Hamilton syringe combined with microsyringe pump was used to deliver 0.5 μl 25 mM AmBleb or vehicle (DMSO) at a rate of 0.5 µl/min. This dose does not cause heart function deviations, because even 0.5 mg systematically introduced AmBleb (130-times higher than the amount injected here) does not affect heart functions (data not shown).

### Measurement of cardiovascular and respiratory functions

Heart rate, pulse distention, breathe rate and blood oxygen saturation was measured by non-invasive MouseOx Plus^R^ Pulse Oximeter for rodents (Starr Life Sciences Corp., PA, USA). 250 g Wistar rats were anesthetized with urethane and Pulse Oximeter Sensor was applied to the shaven neck. 0.5 mg AmBleb dissolved in 200 μl DMSO was injected intraperitoneally and vital functions were followed for more than an hour.

### TTC staining

Prior to our experiments with AmBleb, we checked the reproducibility of the MCAO procedure on 6 animals and determined the extent and the localization of cerebral lesion 24 hours after MCAO. Briefly, we dissected the brain in the coronal plane at the bifurcation of MCA and anterior cerebral artery and visualized the cerebral lesion by TTC staining (0.1 g 2,3,5-triphenyltetrazolium chloride dissolved in 5 ml distilled water).

### Blood perfusion imaging measurements using Single Photon Computed Tomography (SPECT) coupled to neural tissue edema imaging with MRI

Cerebral blood perfusion was measured using the validated and high-sensitivity method of single photon emission computed tomography (SPECT). Brain tissue water content changes and related morphological alterations were regionally assessed by magnetic resonance imaging (MRI) coupled to SPECT. SPECT/MRI images were obtained in each animal at 3 hours, and at 3, 7, 10, 14, 17 and 21 days after the MCAO stroke model induction.

SPECT and MRI were carried out on rats anesthetized with 2% isoflurane in medical oxygen immobilized and fixed in a specifically designed animal anesthesia, restraining and heating bed (Multicell, Mediso Ltd., Hungary). For SPECT detection of blood perfusion changes, 80-160 MBq [^99m^Tc]-HMPAO (Brain-SPECT® kit, Medi-Radiopharma Co Ltd., Hungary) was administered to the animals 60 minutes prior start of SPECT imaging, via tail vein bolus injection in 300-600 µl volumes. The applied radiopharmaceutical, ^99m^Tc-[d,l]-hydroxy-methylene-paraamino-oxime ([^99m^Tc]-HMPAO) accumulates in brain tissue (glia, neurons, microglia, and nonneural cells like choroid plexus) as a function of the tissue blood perfusion in the CNS. After delivery by capillaries, the radiotracer is entrapped inside the cells behind the blood-brain barrier (BBB) and its quantifiable radioactivity is proportional to the initial perfusion of the given brain region.

The SPECT measurements were performed on a NanoSPECT/CT+ (Mediso Ltd, Hungary) imaging system. A helical data acquisition was performed in 50 minutes on each animal with 100 frames and 120 seconds per frame in a 256x256 column/row matrix with 125x125 μm/pixel resolution. Collected data were reconstructed using the Mediso Tera-TomoTM Monte-Carlo model-based maximum likelihood expectation maximization (MLEM) SPECT reconstruction algorithm running on the Nucline (Mediso Ltd., Hungary) software. Brain volumetric radioactivity data in 330 µm isovoxels were obtained and quantified in units of radioactivity measured in MBq/ml.

MRI acquisitions were performed immediately following the [^99m^Tc]-HMPAO injection, using a nanoScan® 1T MRI system (Mediso Ltd, Budapest, Hungary) with a dedicated transmit/receive rat coil. A T2 weighted Fast Spin Echo (FSE) three-dimensional sequence had 460 µm in-plane resolution, slice thickness: 460 µm, repetition and echo times 2000 ms and 78.9 ms, 80 frequency- and 64 phase encodings, 90 degrees flip angle and 2 excitations resulting in a 25 minutes 44 seconds acquisition. Image analysis of 3D SPECT and MRI VOIs was performed with VivoQuant software (InviCRO, Boston, MA). MRI images were employed as reference to the SPECT of the same animal in each imaging time point. During analysis, each animal's SPECT/MRI co-registered image was also co-registered to a MRI brain atlas template using the co-registration tool in SPM12 (7219).

### Quantitative Image Analysis Signal removal from extrameningeal tissue

Firstly, to quantify the MRI and SPECT recordings, the extrameningeal tissues were discarded. Using BrainSuite18a (developed by David Shattuck et al. at UCLA) the skull tissues were removed from the MRI volumes using the skull-stripping pipeline with additional manual alignments in each slice. The final brain masks were applied to the previously co-registered SPECT volumes as well.

### Reconstruction of cerebral edema from MRI images

For the quantification of the cerebral edema volume on the MRI dataset, the pixel intensity histograms of the hemispheres were determined using the stack histogram tool in Fiji 24. For determining the pixel intensity threshold for edema, Gaussian curves were fitted onto the histogram values of the contralesional (right) hemispheres and from the mean of the Gaussian curve; two times the standard deviation was subtracted. This threshold was set as the absolute intensity threshold for the surface tool in the 3D bioimaging and reconstruction software Imaris v 9.0.2. (Bitplane Inc.), to reconstruct the cerebral edema volume. In the contralesional hemisphere, this threshold value excluded 97.6% of all pixels; the remaining 2.4% with the highest pixel intensity correspond to the brain ventricles. The volume of the contralesional ventricles was subtracted from the cerebral edema volume, since the isolation of the ventricles on the edema-affected hemisphere was not possible in most cases. To remove the space-occupying effects of vasogenic edema, we performed a correction method by calculating the differences between the ipsi- and the contralesional hemispheres and removing the excess area from the edema volumes to get the final infarction size 25. For the statistical comparison of edema volumes unpaired t-test was applied.

### SPECT parametrization and segmentation

A parametric map was created from our masked SPECT recordings and the SPECT ^99m^Tc-HMPAO dataset of healthy rats 26, 27 by transforming the SPECT signals to relative rCBF values. In the absence of arterial input function relative rCBF values were used in all analysis, for simplicity throughout the text we refer to relative rCBF as rCBF. For the parametric maps each voxel was transformed sequentially by applying the Lassen's correction algorithm 28 (α = 0.5), selecting the right hemispheres as reference regions. The parametric maps were co-registered with a rat brain atlas 29 and segmented into 72 functional regions described in the atlas (from the 77 regions described in the atlas, regions 4, 13 and 75 did not contain measurement data, regions 56 and 71 contained incomplete data, therefore these were excluded from analysis). The relative rCBF values were determined by averaging the voxel values in ROIs of the co-registered parametric map.

### The rCBF (regional cerebral blood flow) analysis

The co-registered parametric maps were imported to MATLAB R2018b for further analysis:

1. The mean ± SD values of the physiologically normal relative rCBF for each functional brain region were calculated from the dataset of healthy rats. The hypo- and hyperperfused voxels of MCAO treated rats were selected by setting up a threshold for both by subtracting/adding 2xSD to the mean rCBF values of the reference dataset, corresponding to the 95% confidence interval in each individual brain region. Hypo- and hyperperfused brain volumes were constructed by grouping voxels outside these thresholds. 2. We evaluated the relative rCBF changes over time by using the day 0 hypoperfused volume as a 3D mask. The mask was applied to all measurements in the time-series of the same animal and either the voxels contained in these ROIs of the parametric maps were averaged for each time point (voxel-wise), or the SPECT signals were averaged and then transformed using Lassen's algorithm (roi wise). 3. The relative rCBF calculation for the functional regions present in the rat brain atlas was performed for each measurement point by averaging the voxel values corresponding to each region. By relating the values to those of the reference dataset, we obtained discrete relative rCBF values for every functional region. We classified brain regions into two groups based on whether the rCBF value was significantly different (non-regenerated) from the reference regions or not (regenerated).

### Neurological Scoring Tests

To avoid abusing the animals with imaging procedures and neurological test assessments on the same day, neurological scoring tests were carried out 1 day after the imaging days except for day 21. The neurological status of the animals was assessed on days 4, 8, 11, 15, 18 and 21 after MCAO by scoring tests relating to focal and general deficits 30. Slight modifications in the scoring system were implemented as neither epileptic nor circling behavior were present in the animals and climbing was not tested, consequently these three scores were omitted from analysis. The observer who scored the animals was blinded to the identity of the individual animals.

In case of general deficits animals were scored between 0-4 or 0-2 based on the severity as follows: posture (0: normal, 1: hunched, unstable, 2: upright head and/or body rest on ground, 3: lies on side, can assume prone position with strain, 4: passive, lies as placed), spontaneous activity (0: normal, 1: calm, quiet, explores slowly, 2: inert, somnolent, not exploring, 3: lethargic, stuporous, some movements in place, 4: no spontaneous movements), hair (0: normal, 1: localized disorder, especially around eyes and nose, 2: generalized disorder, ruffled and/or dirty fur) and eyes (0: normal, 1: rheumy, 2: dark discharge, 3: semi-closed, 4: closed). Since the condition of eyes was almost completely recovered at day 8 in both control and treated groups therefore it was excluded from the cumulative analysis of general deficit scoring.

In case of focal deficits animals were scored between 0-4 based on the symptoms as follows: body symmetry (0: normal, 1: slight asymmetry, 2: moderate asymmetry, 3: prominent asymmetry, 4: extreme asymmetry), gait (0: normal, 1: stiff, inflexible, 2: limping, 3: trembling, drifting, falling, 4: does not walk), front limb symmetry (0: normal, 1: light asymmetry, 2: marked asymmetry, 3: prominent asymmetry, 4: slight asymmetry, no body/limb movement), compulsory circling (0: not present, 1: tendency to turn to one side, 2: circles to one side, 3: pivots to one side sluggishly, 4: does not advance) and Whisker-response (0: symmetrical response, 1: light asymmetry, 2: prominent asymmetry, 3: absent response ipsilesionally, diminished contralesionally, 4: absent proprioceptive response bilaterally). For analysis, average scores ± SEM were calculated at each time point for the control and treated groups, significance of difference was evaluated by unpaired t-test.

### Cylinder test

To evaluate locomotor asymmetry, MCAO treated rats were tested in an open-top, 21 cm diameter, clear glass cylinder 4, 8, 11, 15, 18 and 21 days after MCAO operation and AmBleb treatment. Forelimb activity was calculated from number of touches of each forelimb against the wall of the arena. Forelimb use was defined as the whole palm touched the sidewall of the cylinder. Number of sidewall touches was counted for 2 minutes.

### Statistics

The number of replicates (n) is indicated in the figure legends and refers to the number of experimental subjects independently treated in each experimental condition. Data are presented as means ± SEM. Statistical analysis was carried out using unpaired two sample t-test in GraphPad Prism 7 and normality was tested using the Shapiro-Wilk test unless otherwise stated. Statistical significance was set at *p < 0.5, **p < 0.01.

## Results

### AmBleb, a potent inhibitor of SMM, relaxes contracted human arterioles

**Figure 1:**
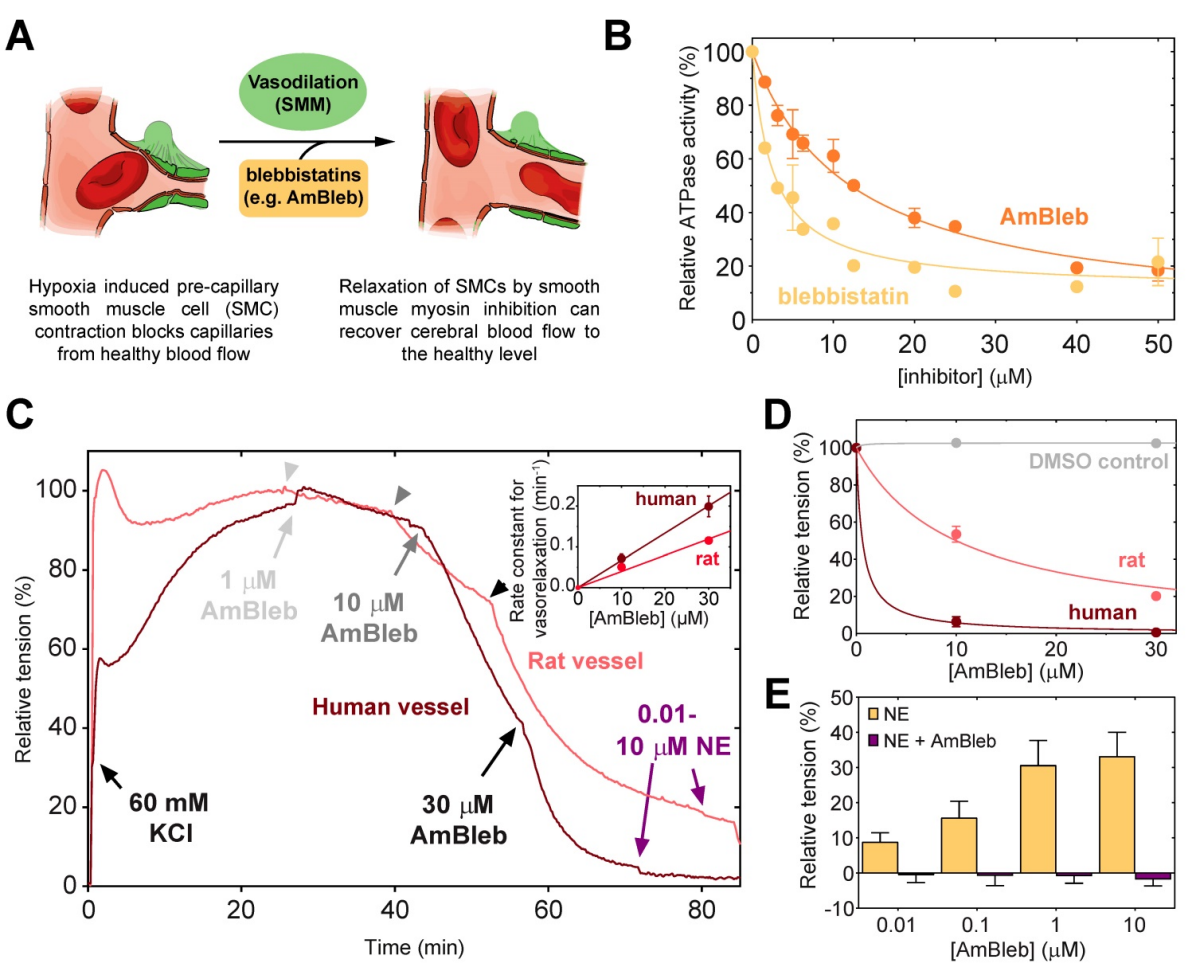
** Direct smooth muscle myosin inhibition relaxes contracted cerebral arterioles. (A)** Schematic figure of inhibition of SMM in pre-capillary smooth muscle cell by AmBleb during stroke. **(B)** ATPase activity of human SMM is efficiently inhibited by blebbistatin and AmBleb (IC50_bleb,SMM_ = 3 µM and IC50_AmBleb, SMM_ = 12 µM). **(C)** AmBleb induced vasodilatation can restore capillary blood flow by relaxing smooth muscle myosin in SMCs as demonstrated on isolated human and rat arterioles. Addition of 1, 10 and 30 μM AmBleb is labeled with light gray, gray and black arrowheads, respectively, series of NE addition (0.01-10 μM) is labeled with purple arrowheads. **(D)** Human arterioles were more sensitive for AmBleb than rat vessels indicated by the faster relaxation rate and the lower ED_50_ values for human arterioles (ED_50,human_< 1 μM, ED_50,rat_= 10 μM), while DMSO alone had no effect on vasodilation.** (E)** The AmBleb-dilated vessels in both human and rat arterioles were unresponsive to norepinephrine (NE), an upstream regulator of vasoconstriction.

Blebbistatin has been previously reported to be an inhibitor of SMM and arterial contractions 31. As vasodilatation through SMC relaxation could be an important effect in stroke therapies (Figure 1A), we assessed that AmBleb retained the inhibitory effects on SMM described for blebbistatin 32, 33 (Figure 1B). The major advantages of AmBleb are its improved solubility, chemical stability and non-cytotoxicity in contrast to blebbistatin 15. Therefore, AmBleb can be safely applied *in vivo*. We measured the vasodilatation effect of AmBleb on isolated rat and human arterioles (Figure 1C-E**,** Figure S1). Contracted arterioles were treated with increasing amounts of AmBleb and full dilatation of the arterioles was achieved at 30 μM AmBleb concentration with human arterioles being more responsive to AmBleb treatment than rat vessels (Figure 1D-E). Importantly, the upstream regulator of vasoconstriction, norepinephrine, did not cause vessel constriction in the AmBleb-dilated arterioles demonstrating that direct myosin inhibition overcomes signaling from upstream regulators (Figure 1E). These *in vitro* results encouraged us to investigate the effects of AmBleb in *in vivo* rodent stroke models.

### Substantial improvement in post-stroke cerebral blood flow by AmBleb treatment

**Figure 2:**
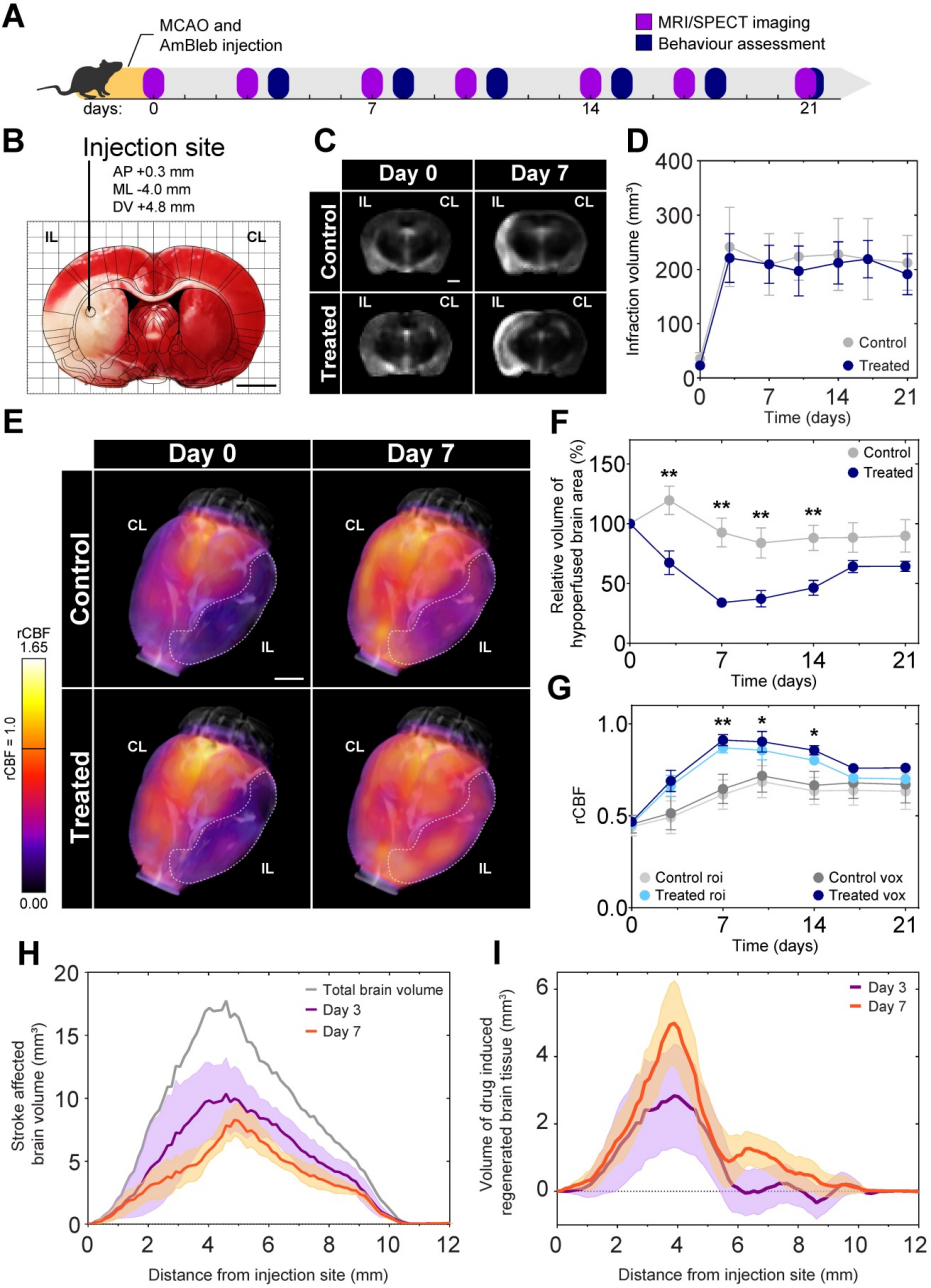
** Cerebral blood flow at the ischemic brain region recovers 7 days after MCAO while brain edema remains constant during the 3 weeks of investigation. (A)** Experimental timeline. **(B)** TTC staining of a representative brain slice shows a well-defined ischemic region as a result of MCAO. The injection site is marked on the overlaid rat brain atlas. **(C)** Representative MRI slices of brain edema development of control and AmBleb treated animals' brains obtained 3 hours (day 0) and 7 days after MCAO. **(D)** Quantitative MRI analysis of brain edema development of control and AmBleb treated animals' brains up to 21 days after MCAO. **(E)** 3D reconstruction of rCBF values in the ischemic area. Dotted line on the 3D reconstruction images marks the hypoperfused volume at day 0 and day 7. **(F)** Hypoperfused volumes at each time point up to 21 days. In the relative hypoperfused volumes significant changes can already be observed between the treated and control animals from day 3. **(G)** rCBF values in the volume defined as hypoperfused at day 0 on the ipsilesional hemisphere both voxel-wise (dark blue/grey) and roi-wise (light blue/grey). **(H)** Volume of brain tissue significantly different from the healthy level is shown as a function of distance from AmBleb injection site on day 3 (purple) and day 7 (orange). Total brain volume is also shown (gray) for comparison. **(I)** Difference of healthy brain tissue volumes between treated and control brains is shown as a function of distance from AmBleb injection site on day 3 (purple) and day 7 (orange). Note that AmBleb effect is the most pronounced in the penumbra region (2-6 mm from the injection site), which has high importance in stroke interventions. Data represent the mean ± SEM (n=6 per group), p values were determined by unpaired t-test. *p < 0.05, **p < 0.01, scale bars: 2.5 mm.

As a proof-of-concept for the positive effects of direct myosin-2 inhibition on recovery after stroke, MCAO was applied to model acute ischemic stroke on 34 male Wistar rats. To maximize reproducibility we did not include females, as sex may influence the response to neuroprotective agents 34. A single injection of AmBleb or DMSO vehicle was administered directly into the ischemic region with stereotaxic targeting immediately after the removal of the occluding filament to mimic the effect of a potential stroke treatment following thrombectomy (Figure 2A-B). Three animals died within 72 hours, the remaining 31 were monitored for 21 days. We investigated the development of brain edema and cerebrovascular blood flow by MRI and SPECT, respectively, on 6 control and 6 AmBleb treated animals for 3 weeks after the MCAO operation.

We also investigated the effect of AmBleb treatment on cardiovascular and respiratory functions (Figure S2). We administered intraperitoneally 125-times higher dose (0.5 mg) AmBleb to the same size Wistar rats than those underwent MCAO operation and AmBleb treatment (4 μg). Importantly, we did not see any significant change on heart rate, blood flow through the carotid arteries, breath rate and oxygen saturation, suggesting that even if AmBleb is not selective for SMM, it does not influence vital functions in the applied concentration range.

Brain tissue edema was identified on the skull-stripped MRI images based on the intensity histograms of the two hemispheres. In agreement with other studies 35-37, we observed brain tissue edema development 3 days after MCAO (Figure 2C), which remained consistent during the 21 days of investigation both in the control and in the AmBleb treated animals (Figure 2D). The lack of decrease in brain edema size during a 3-week follow-up period is also in line with recent findings by other groups 38-46, and due to its different molecular targets AmBleb is not expected to play a role edema development 47. Nonetheless, a drastic increase in cerebral blood flow was detected by SPECT in the AmBleb treated animals compared to the control group (Figure 2E). Relative regional cerebral blood flow (rCBF) was determined from SPECT records by the Lassen's correction algorithm 28, 48 and the rCBF was compared to those of 15 healthy Wistar male rats 26, 27 (Figure S3A).

We followed the change of the total hypoperfused volume in the treated and untreated animals. After 3 days of MCAO, a striking difference was observed between the control and the AmBleb treated animals and the difference remained significant for another 11 days (Figure 2F). A slight but statistically not significant relapse of the hypoperfused volume was detected after day 14 in the AmBleb treated group.

We also determined the changes in rCBF values in the initially hypoperfused region for 21 days (Figure 2G). By day 7, rCBF values in the stroke affected hypoperfused region were 41% higher in the AmBleb treated animals than those of the control rats. This difference in blood flow remained significant for 1 week and then a slight relapse was observed. We note that the rCBF averaging based on ROI and voxels gave similar results (Figure 2G).

From the total number of voxels that are significantly different from the healthy counterparts we could define the stroke-affected brain volume as a function of distance from the injection site (Figure 2H). These values were calculated for both the control and the AmBleb treated animals from which we could determine brain volumes that were recovered by AmBleb treatment over the control recovery level (Figure 2I). This analysis shows the core region, which is prone to cell death (0-1 mm) could not be recovered effectively by AmBleb. However, the penumbra region (2-6 mm from the injection site) improved drastically better than in the control group.

**Figure 3:**
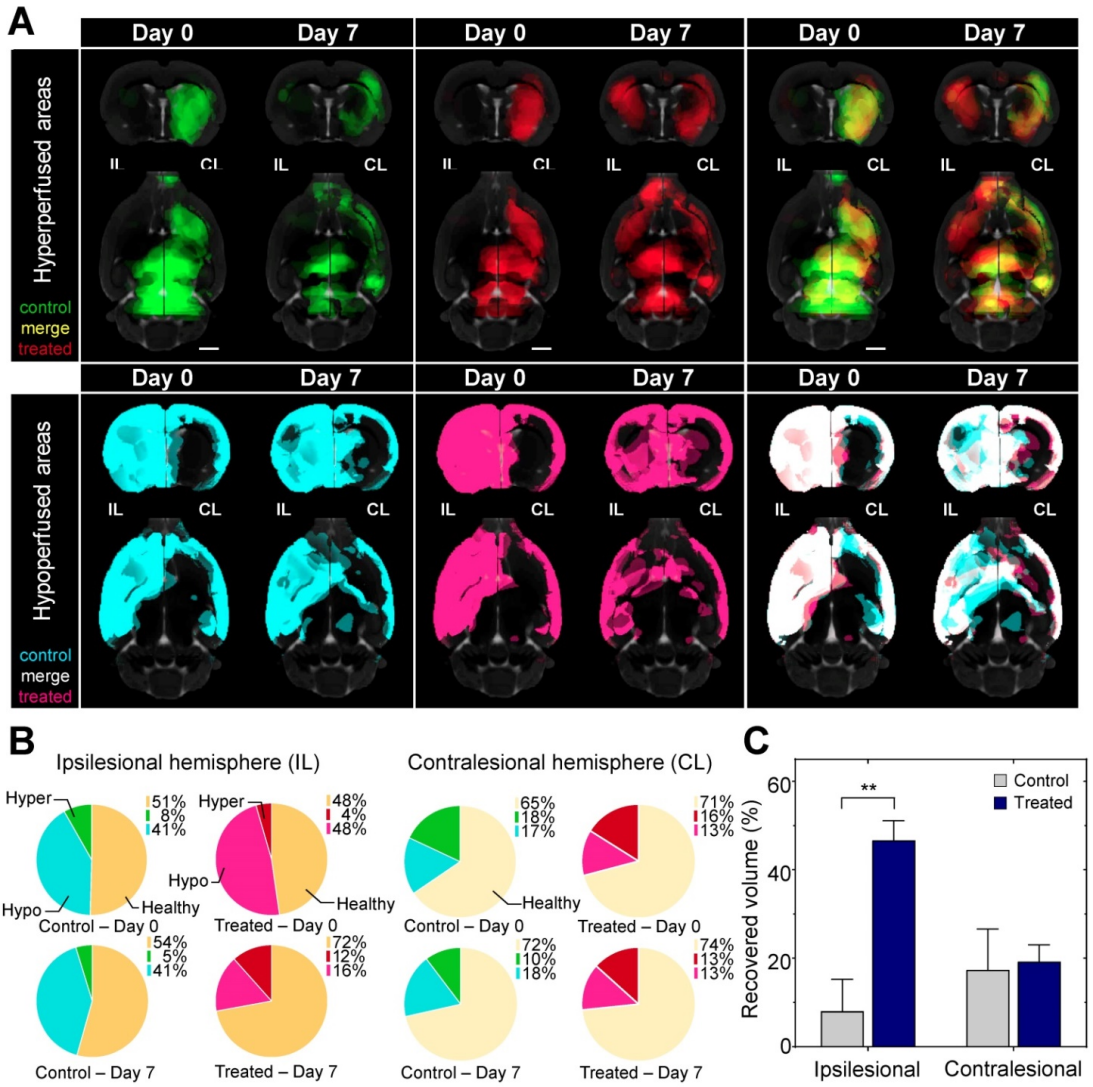
** Hypoperfused brain areas are significantly reduced in the ipsilesional hemisphere upon AmBleb treatment. (A)** Extended brain slices: (15 mm) showing hypo- (cyan or pink) and hyperperfused (green or red) areas co-registered with MRI in control or treated groups, respectively. Images were created by averaging the parametric maps containing only the hypo- and hyperperfused voxels. On day 0 control and treated groups were identically affected by MCAO indicated by the substantial overlap of hypo- and hyperperfused areas on the merged images on the right panels (cf. Table S1). By day 7 on the ipsilesional hemisphere in the treated group greater volume was hyperperfused and less was hypoperfused compared to the control group. Scale bar: 2.5 mm.** (B)** Graphical representation of the fraction of healthy (orange), hypo- (cyan in control, pink in treated animals) and hyperperfused (green in control, red in treated animals) brain regions at day 0 and day 7 in the ipsilesional (upper four pie charts) and contralesional (lower four pie charts) hemispheres. All data points in this figure are from Table S1. (n=6 for each group).** (C)** On day 7 the affected volume in control (gray bars) and treated (blue bars) animals decreased markedly. At the ipsilesional side 46.7% of originally affected areas recovered in the treated animals while only 8% of the affected areas improved in control rats. Blood flow changed identically on the contralesional hemispheres of control and treated animals.

By day 7 in the ipsilesional hemisphere the hypoperfused volume shrank by 67% in the treated, while only 1% in the untreated animals. In the contralesional hemisphere the volume of the hypo- and hyperperfused areas did not change significantly in the treated and the untreated animals indicating direct local effect of AmBleb (Figure 3, Table S1).

### Cerebral blood flow of 32 out of 65 affected brain regions in the AmBleb treated animals was restored to healthy levels

**Figure 4:**
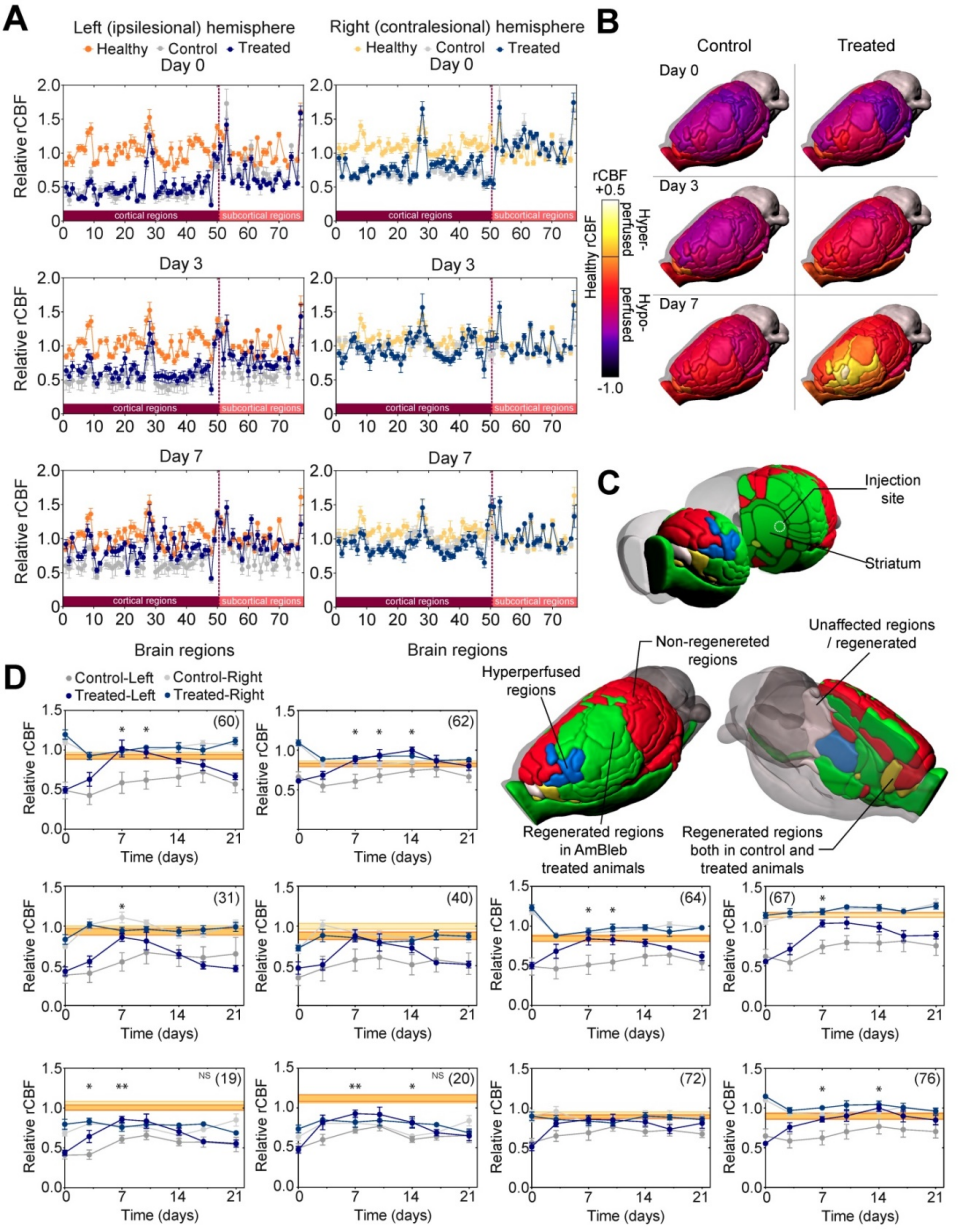
** Cerebral blood flow in 32 out of 65 affected brain regions significantly improved in the ipsilesional hemisphere 7 days after stroke. (A)** Healthy (orange), control (gray) and treated (dark blue) rCBF levels in 72 functional regions (50 cortical and 22 subcortical) of both hemispheres on day 0, 3 and 7 after MCAO. On the ipsilesional hemisphere, treated and control rCBF values overlap almost entirely on day 0. However, on day 7 the control and the AmBleb treated regions strikingly differ. Difference between control and AmBleb treated rCBF in the contralesional hemisphere stays very low (c.f. Figure S1). **(B)** rCBF values of 72 functional regions in the ipsilesional hemisphere of the brain in the control (left panels) and AmBleb treated group (right panels) compared to the reference healthy rCBF level. Color-coding reflects the rCBF values relative to the rCBF of the respective region in healthy animals (c.f. Figure S1B). Orange (healthy rCBF value) represents that the rCBF is identical to that of the healthy animals. Note that cerebellum was excluded from the analysis. **(C)** Color-coding reflects rCBF improvement from day 0 to day 7 in each functional region of the ipsilesional hemisphere. Gray regions recovered only in the control or were unaffected by MCAO. AmBleb treatment resulted in full recovery (green) or hyperperfusion after recovery (blue). Yellow regions improved in both the control and treated groups. Red regions did not recover in any group. **(D)** Time dependent changes in rCBF values of key functional regions (striatum - 60, internal capsule - 62, primary somatosensory cortex barrel field - 31, secondary somatosensory cortex - 40, pallidum - 64, corpus callosum - 67, primary motor cortex - 19, secondary motor cortex - 20, olfactory structures - 72, optic pathways - 76). Each panel represents the rCBF values of the ipsilesional (left, darker symbols) and contralesional (right, lighter symbols) hemispheres of the control (gray) and the AmBleb treated (blue) animals with reference to the physiologically normal rCBF levels of healthy animals (orange bands) in the indicated brain regions. Data represent the mean ± SEM (n=15 AmBleb treated, n=16 control) *p < 0.05. ^NS^ marks motor cortex regions where rCBF values remain significantly below the healthy range on day 7, however, still show marked improvements between day 0 and day 7. Data represent mean ± SEM (n=6 control/AmBleb or 15 healthy).

In healthy animals there are both characteristic differences in rCBF levels between the functional regions within a hemisphere, and also region-specific differences between the two hemispheres (Figure 4A, Figure S3). To identify functional brain regions that are affected by stroke and those brain regions that are prone to regeneration, the rCBF values of 72 functional brain regions were compared 29 (Table S1) in both hemispheres of the control and AmBleb treated animals to those 15 healthy Wistar male rats 26, 27 (Figure 4A, Figure S3). On Day 0 (3 hours after MCAO) the rCBF values of control and treated groups showed no difference; rCBF values in the ipsilesional hemisphere and the contralesional cortex regions were generally lower, while rCBF values in the contralesional subcortical regions were slightly higher compared to the healthy levels. Although the rCBF values had a tendency to approach healthy levels during the first week in both groups, recovery in the ipsilesional hemisphere was enhanced by AmBleb treatment (Figure 4B). On day 0, MCAO affected the rCBF of 65 and 67 regions in the control and treated animals, respectively. (Figure 4B, Figure S3-5, Table S2). On day 7, 32 out of the 65 affected areas recovered in the treated animals while only 8 out of the 67 affected regions showed improvement in control animals. (Figure 4C, Figure S3-5, Table S2). 5 of these regions improved both in the control and treated animals while 27 functional regions recovered only in the AmBleb treated animals (Figure 4C). 3 recovered hypoperfused regions in the treated animals became slightly hyperperfused (regions 33, 36 and 61), while none of them became hyperperfused in the control group. AmBleb treatment significantly enhanced the recovery as the rCBF of 32 regions including the most important functional regions were recovered (Figure 4D, Figure S4). By contrast, these regions had significantly reduced rCBF values in the control animals. We note that the rCBF values of some important regions responsible for controlling motor functions did not recover to the normal range, however, their rCBF values showed marked improvements between day 0 and day 7 in treated animals (Figure 4D). We also note that the functional regions closer to the injection site had a higher tendency for improvement than those farther away from the injection site (Figure 4C), also indicating the direct, local effects of AmBleb in the recovery of stroke-related ischemic brain area.

### Neurological deficits significantly improved in AmBleb treated rats

**Figure 5:**
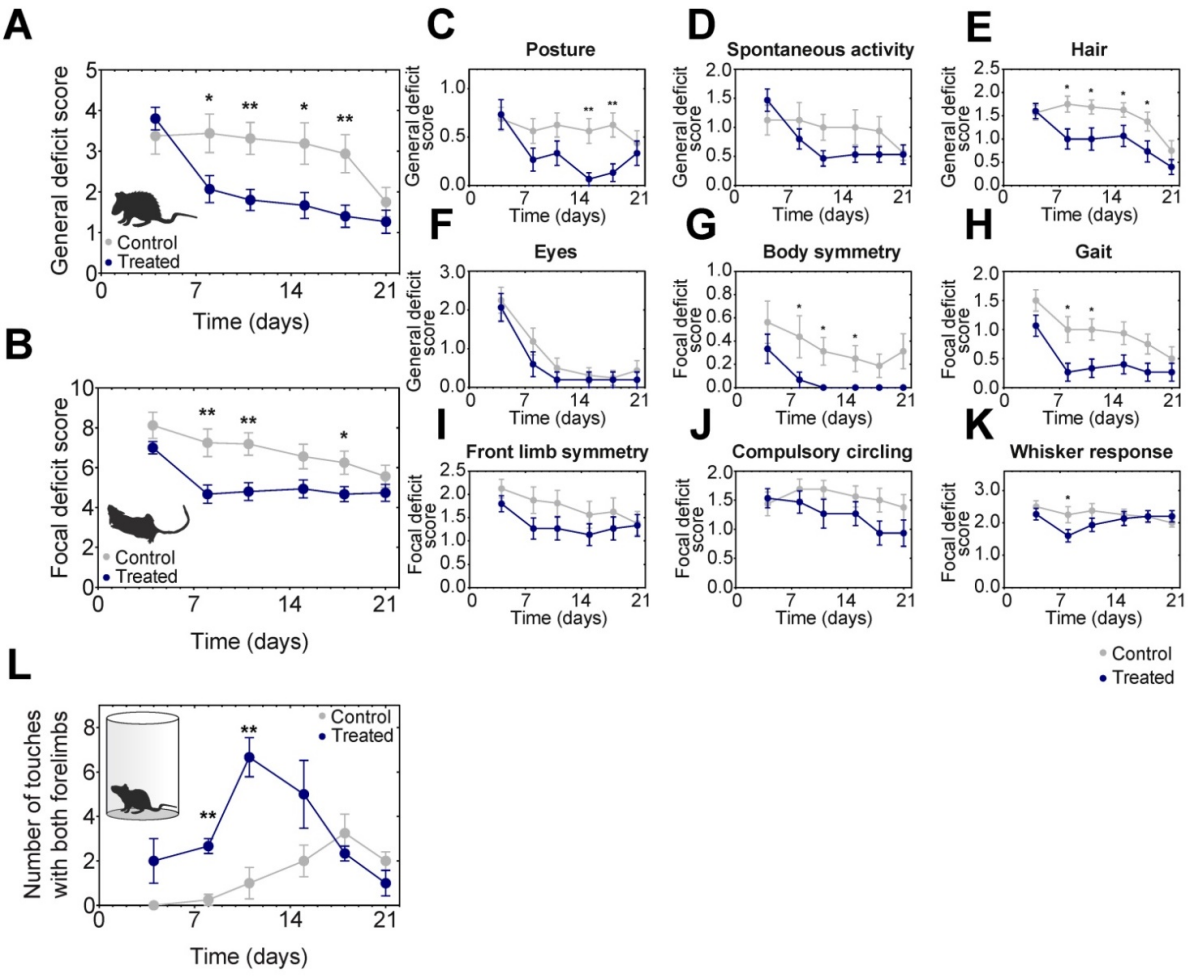
** Neurological deficits improve significantly faster in AmBleb treated animals compared to the control group.** Cumulative analysis of general **(A)** and focal **(B)** deficit scores indicates significant difference between control and treated animals during the test period. Average scores of individual symptoms illustrate the details underlying the improvement in the neurological status **(C-K)**. Cylinder test was conducted to investigate the locomotor assessment and significant improvement was observed between day 8 and day 11 **(L)**. Data represent the mean ± SEM (n=15 AmBleb treated, n=16 control) *p < 0.05, **p < 0.01.

After MCAO the general and focal neurological deficits were followed on 16 control and 15 AmBleb treated animals including the ones tested in MRI/SPECT imaging (Figure 5A-K). Locomotor asymmetry was assessed in a cylinder test on 4-4 animals and significant improvement was observed on days 8-11 (Figure 5L). General deficit scoring was performed on body posture, spontaneous activity, hair ruffle and eye conditions (Figure 5C). Since eye condition recovered almost completely by day 8 in both groups, these scores were not cumulated with the other three parameters. These three deficits improved substantially in the treated animals by day 8. The difference of the general deficits cumulative scores remained significant between the treated and control groups for 18 days (Figure 5A).

We further analyzed focal deficits of the animals in body symmetry, gait, front limb symmetry, compulsory circling and Whisker response (Figure 5B). Cumulative focal deficit scores were significantly lower in treated animals than in control ones from day 8 throughout the entire test period except for the last day (Figure 5B). Interestingly, except for body posture and Whisker response, we did not observe the relapse in general and focal neurological test features that we observed in blood flow analysis (cf. Figure 2), indicating that AmBleb treatment might have different dynamic effects on vascular and neurological functions.

## Discussion

We demonstrated that AmBleb has drastic effect on vasodilation of both rat and human arterioles due to the effective relaxation of SMM in the same concentration range as the inhibitory constant for myosin-2 ATPase activities. We propose that the effective vasodilatator effect is realized in the *in vivo* experiments, and AmBleb treatment significantly enhanced the rCBF and accelerated neurological improvements after MCAO in the 3-week follow-up period. The observed changes in rCBF values and neurological scores of the control group are in line with previous studies 49, 50 confirming the reliability of our tests.

Although AmBleb is not myosin-2 isoform specific, the locally applied effective dose is more than 100-times less than the dose administered systematically with no effect on cardiovascular and respiratory functions (Figure S2), suggesting that effective treatment can be achieved with no effect on vital functions.

**Figure 6:**
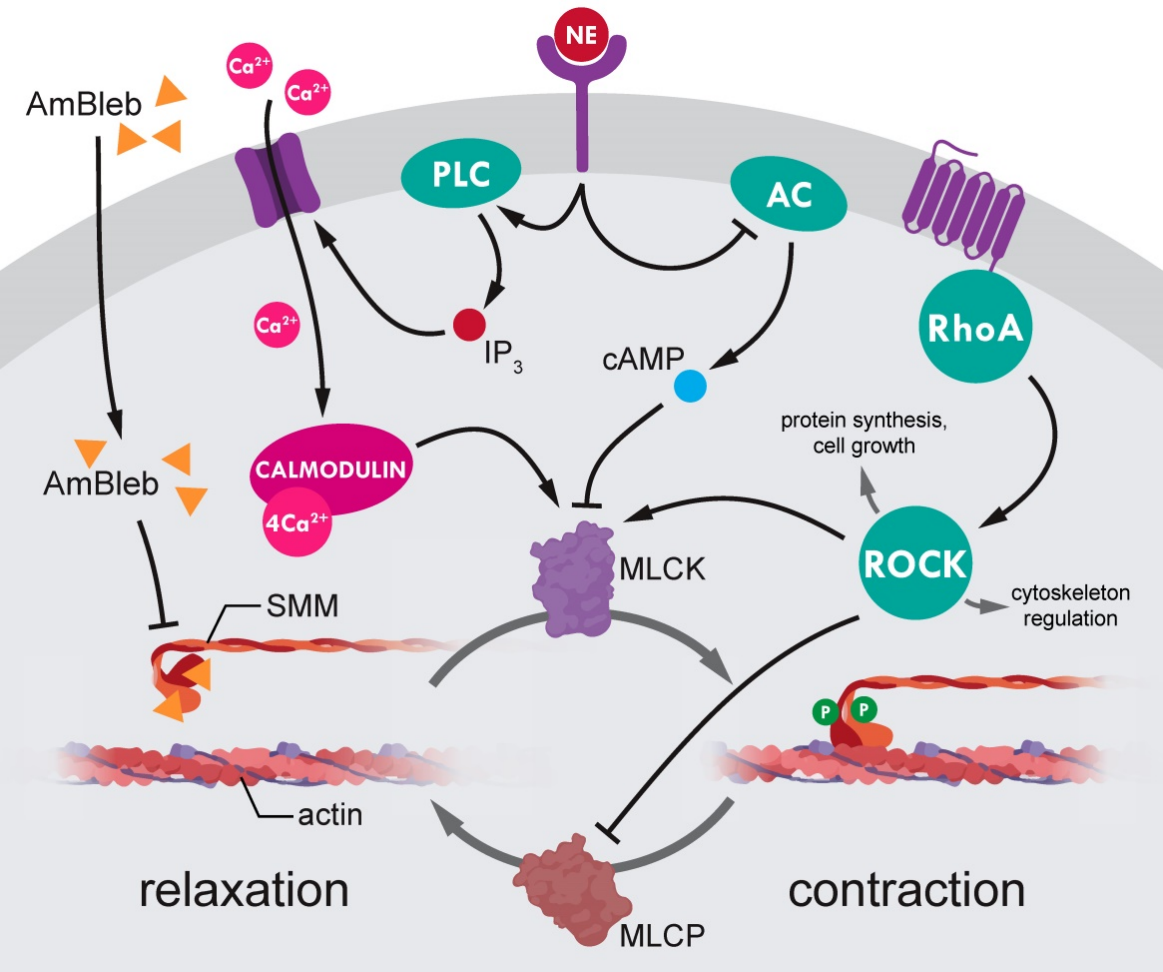
** Regulation of smooth muscle myosin activity in the precapillary smooth muscle cells.** SMM is the final effector of small vessel constriction. Actomyosin contraction is regulated through dynamic phosphorylation pattern of myosin light-chains mediated by myosin light-chain kinases (MLCK) and phosphatases (MLCP). Both MLCK and MLCP are under the complex control of several upstream regulators (e.g. Rho-associated kinase (ROCK) and norepinephrine (NE)). However, ROCK is a central hub protein, which modulates essential cellular processes such as protein synthesis, cell growth and cytoskeleton regulation, thus, ROCK inhibition to achieve SMC relaxation would lead to wide range of side effects. By contrast, direct inhibition of myosin by AmBleb (yellow triangles) can relax SMC contraction independently from upstream signaling pathways as we demonstrated in Figure 1 that AmBleb induced actomyosin relaxation could not be overcome by vasoconstriction signaling from NE pathway.

Our advance in improving recovery after stroke by direct SMM inhibition provides a new concept that earlier successes in MCAO post-stroke regeneration with ROCK pathway inhibitors 14, 51-59 may, at least partly, also involve SMM as main downstream effector (Figure 6). The ROCK inhibition mediated SMM inhibition, and the corresponding opening of capillaries might have been a major effect of liposomal fasudil treatment 14. However, inhibition of regulatory hub proteins upstream of myosin may also have negative effects on stroke regeneration by inducing large scale actin depolymerization 11 and inducing apoptotic pathways 60. Hence direct inhibition of SMM could be of substantial value to achieve the same positive effects as ROCK inhibition while avoiding the unwanted negative side effects. The importance of direct inhibition of SMM is even highlighted that the upstream regulator norepinephrine did not induce vessel constriction in the AmBleb-dilated arterioles (Figure 1C, 1E, Figure 6).

Direct myosin-2 inhibition could also affect other substantial processes during stroke regeneration, which might also contribute to improved rCBF values after AmBleb treatment. It was demonstrated that myosin-2 inhibition promotes angiogenic sprouting and initial vascular branching 61. The Rho/ROCK signaling pathway, which is the upstream regulator of SMM activity, has also been identified as an important regulator of endothelial permeability, and consequently direct myosin-2 inhibition has been supposed to play an important role in maintaining endothelial integrity and vascular permeability 62, 63. Furthermore, blebbistatin was shown to decrease the contractile force of platelets which has an important role to hold the integrity of homeostatic clot 64, suggesting that myosin-2 inhibition can also help to remove the blood clots formed during ischemia.

The presented *in vivo* results provide a proof-of-concept and encourage the development of a translationally more relevant administration of the drug and further tests on more complex disease models, such as animals with cardiovascular diseases. In addition, the combination of AmBleb with edema reducing therapies could have the potential to extend the efficiency of the treatment for ischemic stroke and hence improve the neurological outcomes 10, 11, 14, 51-59, 65-68.

We established an unbiased method to assess the effects of MCAO and the dynamics of recovery in a large number of hypoxia-affected brain regions using published reference values of healthy rats. By using an independent reference set instead of comparing the ipsilesional and contralesional hemispheres, we were able to define the affected regions on both hemispheres after MCAO and found substantial improvement in the ipsilesional side of the AmBleb treated group. As previously shown, ischemia-induced hypoperfusion may affect certain brain regions differently based on the cerebral blood flow reduction, the degree of collateral circulation and the metabolic activity in human patients 69. After 14 days of the treatment we observed a tendency to relapse in both the rCBF values and hypoperfused brain volumes, which could possibly be due to the single administration of AmBleb and the additional effect of the permanent edema observed in the MRI images. In contrast, the relapse was not obvious in the neurological deficit scores. Moreover, several neurological features improved substantially when rCBF values were in the relapsing phase.

We also showed that direct targeting of the effector molecule, SMM, has the same positive consequence but without the possible broad side-effect profile emerging from the inhibition of a central regulatory hub kinase, ROCK. The effects of the pharmacologically safe myosin-2 inhibitor, AmBleb indicate that SMM is a promising drug target candidate in ischemic stroke treatment. Thus, further studies are encouraged to assess the effect of myosin-2 inhibition on regeneration after stroke involving fine sensorimotor and cognitive tests on older animals especially with comorbidities to more prudentially approach the human risk conditions according to the current consensus-based guidelines in stroke research 70.

## Conclusion

Direct targeting of SMM in precapillary SMCs and consequent opening of permanently blocked capillaries in the ischemic brain regions may fulfill both short- and long-term requirements towards an innovative medication. In addition, its combination with the current treatments has the potential to extend the treatment-eligibility criteria thereby allowing effective therapies to a broader population of stroke patients.

## Supplementary Material

Supplementary figures and tables.

## Abbreviations

AmBleb: *para*-aminoblebbistatin
MCAO: middle cerebral artery occlusion
MRI: magnetic resonance imaging
ROCK: Rho-associated kinase
rCBF: relative regional cerebral blood flow
SMC: smooth muscle cell
SMM: smooth muscle myosin
SPECT: single photon emission computed tomography
