# Supplementary material for: Direct myosin-2 inhibition enhances cerebral perfusion resulting in functional improvement after ischemic stroke
Source: Theranostics. 2020 Apr 6;10(12):5341–56. doi: 10.7150/thno.42077 (PMC7196296; doi:10.7150/thno.42077)
Supplement: Supplementary file 1 — Supplementary figures and tables. [file thnov10p5341s1.pdf]

Supplementary Materials

for manuscript entitled 'Direct myosin-2 inhibition enhances cerebral perfusion resulting in functional improvement after ischemic stroke'

by Máté Pénzes#, Demeter Túrós#, Domokos Máthé, Krisztián Szigeti, Nikolett Hegedűs, Anna Ágnes Rauscher, Péter Tóth, Ivan Ivic, Parasuraman Padmanabhan, Gabriella Pál, Árpád Dobolyi, Máté Gyimesi and András Málnási-Csizmadia*

Supplementary Tables

	Healthy volume (mm^3)	Affected volume (mm^3)	Affected volume (% of total)	Recovered volume (% of affected at Day 0)	Hypoperfused volume (mm^3)	Fraction of affected area (%)	Hyperperfused volume (mm^3)	Fraction of affected area (%)
**Ipsilesional hemisphere (Total volume = 957.9 mm^3)**								
**Control**								
**Day 0**	483.1	474.8	49.6%		395.9	83.4%	78.9	16.6%
**Day 7**	521.1	436.8	45.6%	**8%**	391.4	89.6%	45.4	10.4%
**Treated**								
**Day 0**	457.2	500.7	52.3%		457.8	91.4%	42.9	8.6%
**Day 7**	690.9	267.0	27.9%	**46.7%**	155.9	58.4%	111.1	41.6%
**Contralesional hemisphere (Total volume = 960.9 mm^3)**								
**Control**								
**Day 0**	629.3	331.6	34.5%		158.5	47.8%	173.1	52.2%
**Day 7**	686.9	274.0	28.5%	**17.4%**	174.8	63.8%	99.2	36.2%
**Treated**								
**Day 0**	681.4	297.6	29.1%		125.1	44.7%	154.5	55.3%
**Day 7**	735.2	225.8	23.5%	**19.2%**	112.8	50.0%	113.0	50.0%

Table S1. Analysis of affected brain areas on both hemispheres upon AmBleb treatment. Numerical analysis of averaged ipsi- and contralesional hemispheres of control and AmBleb treated brains. Graphical representations of these results are shown in Figure 3.

	Area No.	**Control**		**Treated**	
Retrosplenial Granular Cortex, b region	28	+	-	+	+
Substantia Nigra	54	+	+	+	+
Pineal Gland	77	+	+	+	+
Primary Somatosensory Cortex, oral dysgranular zone	33	-	+	+	-
Frontal Assocn Cortex	49	-	+	+	+
Midbrain	53	-	+	+	+
Pituitary	74	-	+	+	+
Thalamus	61	+	+	-	-
Agranular Insular Cortex, posterior part	2	-	-	-	-
Primary Auditory Cortex	5	-	-	-	-
Secondary Auditory Cortex, dorsal area	6	-	-	-	-
Secondary Auditory Cortex, ventral area	7	-	-	-	-
Cingulate Cortex, area 2	9	-	-	-	-
Dorsal Intermediate Entorhinal Cortex	11	-	-	-	-
Dorsolateral Entorhinal Cortex	12	-	-	-	-
ectorhinal cortex	14	-	-	-	-
frontal cortex, area 3	15	-	-	-	-
lateral parietal association cortex	18	-	-	-	-
primary motor cortex	19	-	-	-	-
secondary motor cortex	20	-	-	-	-
medial entorhinal cortex	21	-	-	-	-
perirhinal cortex	23	-	-	-	-
parietal cortex, posterior area, caudal part	24	-	-	-	-
parietal cortex, posterior area, rostral part	26	-	-	-	-
retrosplenial dysgranular cortex	27	-	-	-	-
primary somatosensory cortex	30	-	-	-	-
primary somatosensory cortex, hindlimb region	35	-	-	-	-
temporal associatin cortex	41	-	-	-	-
primary visual cortex	42	-	-	-	-
primary visual cortex, binocular area	43	-	-	-	-
primary visual cortex, monocular area	44	-	-	-	-
secondary visual cortex, lateral area	45	-	-	-	-
secondary visual cortex, mediolateral area	46	-	-	-	-
ventral intermediate entorhinal cortex	48	-	-	-	-
cingulum	52	-	-	-	-
diagonal domain	58	-	-	-	-
hippocampal formation	63	-	-	-	-
amygdala	68	-	-	-	-
isocortex	70	-	-	-	-
primary somatosensory cortex, jaw region	36	-	+	-	-
retrosplenial granular cortex, c region	29	+	-	-	+
agranular insular cortex, dorsal part	1	-	-	-	+
cingulate cortex, area 1	8	-	-	-	+
dysgranular insular cortex	10	-	-	-	+
granular insular cortex	16	-	-	-	+
granular insular + dysgranular insular cortex	17	-	-	-	+
medial parietal association cortex	22	-	-	-	+
parietal cortex, posterior area, dorsal part	25	-	-	-	+
primary somatosensory cortex, barrel field	31	-	-	-	+
primary somatosensory cortex, dysgranular zone	32	-	-	-	+
primary somatosensory cortex, forelimb region	34	-	-	-	+
primary somatosensory cortex, shoulder region	37	-	-	-	+
primary somatosensory cortex, trunk region	38	-	-	-	+
primary somatosensory cortex, upper lip region	39	-	-	-	+
secondary somatosensory cortex	40	-	-	-	+
secondary visual cortex, mediomedial area	47	-	-	-	+
septum	57	-	-	-	+
hypothalamus	59	-	-	-	+
striatum	60	-	-	-	+
internal capsule	62	-	-	-	+
pallidum	64	-	-	-	+
fimbria	66	-	-	-	+
corpus callosum	67	-	-	-	+
preoptic area	69	-	-	-	+
olfactory structures	72	-	-	-	+
bed nucleus of the stria terminalis	73	-	-	-	+
optic pathways	76	-	-	-	+
agranular insular cortex, ventral part	3	-	+	-	+
prelimbic cortex IL infralimbic cortex	50	-	+	-	+
Medial, ventral, orbital, lateral a dorsolateral orbital cortex	51	-	+	-	+
anterior commissure	55	-	+	-	+
accumbens nucleus	65	-	+	-	+

Table S2. – Complete list of brain regions affected in rat MCAO stroke model. rCBF values of 72 different functional regions in the brain were analyzed and compared to the reference healthy dataset. Brain regions with negative symbol (-) show statistically significant differences compared to the reference regions while plus symbol (+) marks regions, which do not possess significantly different rCBF values compared to the reference dataset.

Supplementary Figures

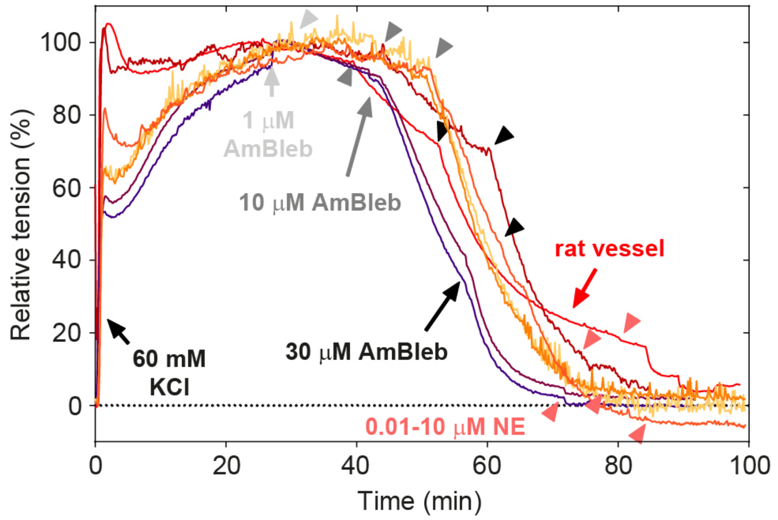

Figure S1. – AmBleb induced vasodilatation of cerebral arterioles from human and rat brain samples. Cerebral arterioles were treated with KCl in a wire-myograph to induce vasoconstriction. After reaching the fully contracted state 1 µM (light gray arrows), 10 µM (gray arrows) and 30 µM (black arrows) AmBleb was added to the vessels and vasodilatation was followed by the reduced tension of arterioles. Note that human vessels responded to AmBleb with faster and larger extent of vasodilatation than the rat vessels. Moreover – as also shown in Figure 1C – series of NE addition (0.01-10 µM, labeled with pink arrowheads) could not contract the AmBleb relaxed arterioles suggesting that direct decoupling of the contractile acto-myosin system hinders cells to respond to upstream constricting signals (c.f. Figure 6).

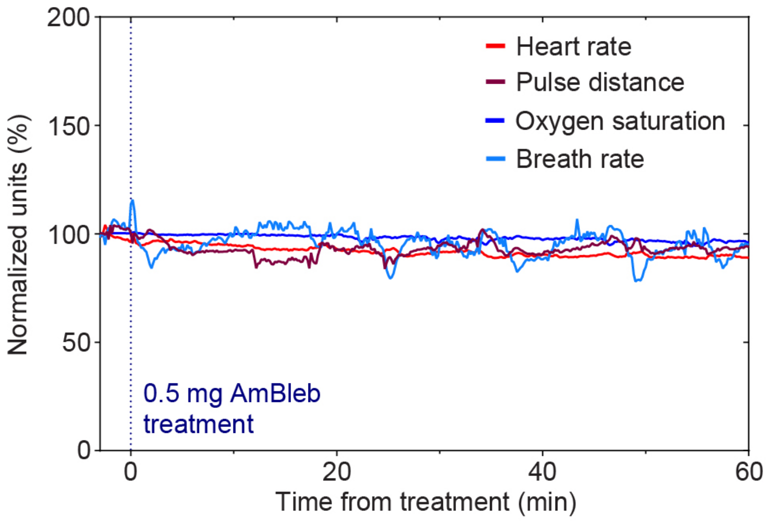

Figure S2. – Cardiovascular and respiratory functions of rats after systemic treatment with 0.5 mg AmBleb. AmBleb is not selective for SMM, thus it may affect heart and respiratory functions due to inhibition of skeletal and cardiac myosin-2 isoforms. Therefore, we tested if systemic treatment of Wistar rats with 0.5 mg AmBleb, which is 125-times higher than the dose used in local injection after MCAO, could have adverse effects on vital functions. Importantly, both cardiac (heart rate (red), pulse distance (purple)) and respiratory (breath rate (light blue), oxygen saturation (blue)) functions were practically unaffected 1 hours after AmBleb treatment, which is 4-times higher time window than that required to see adverse changes with higher AmBleb doses (data not shown). These results suggest that the effective AmBleb dose used in local treatment is safe for the animal.

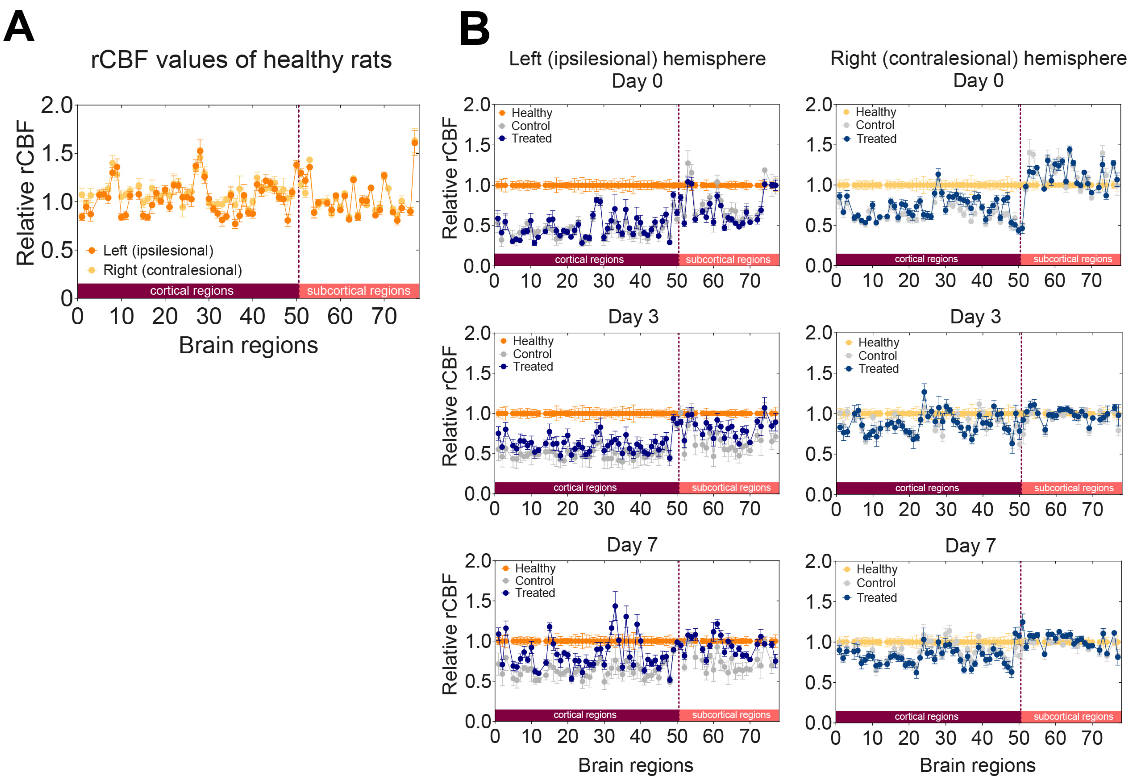

Figure S3. – rCBF values of 72 regions on the two hemispheres of healthy and treated Wistar male rats. (A) The average rCBF values for each brain region of 15 healthy Wistar rats. The rCBF values in the left/ipsilesional (dark orange) and the right/contralesional (light orange) hemispheres correlate with each another in the subcortical regions. In some cases, significant difference could be detected between the left and the right hemispheres: in the insular cortex regions (No.1 and No.10) and in the primary somatosensory cortex regions (No.33 and No.34). Moreover, drastic differences could be observed between brain regions: rCBF in the retrosplenial granular cortex (No. 28) and the pineal gland (No.77) was almost two-fold higher than that in the amygdala (No. 68) and the preoptic area (No.69). Regional names and their serial numbers can be found in Table S2. **(B)** The graphs depict the same data set as in Figure 4A, where the healthy (orange), the control (gray) and the AmBleb treated (blue) rCBF levels are shown in all 72 functional regions except that the rCBF levels are normalized to the healthy rCBF level for each functional region. Color-coding on Figure 4B is based on these calculations.

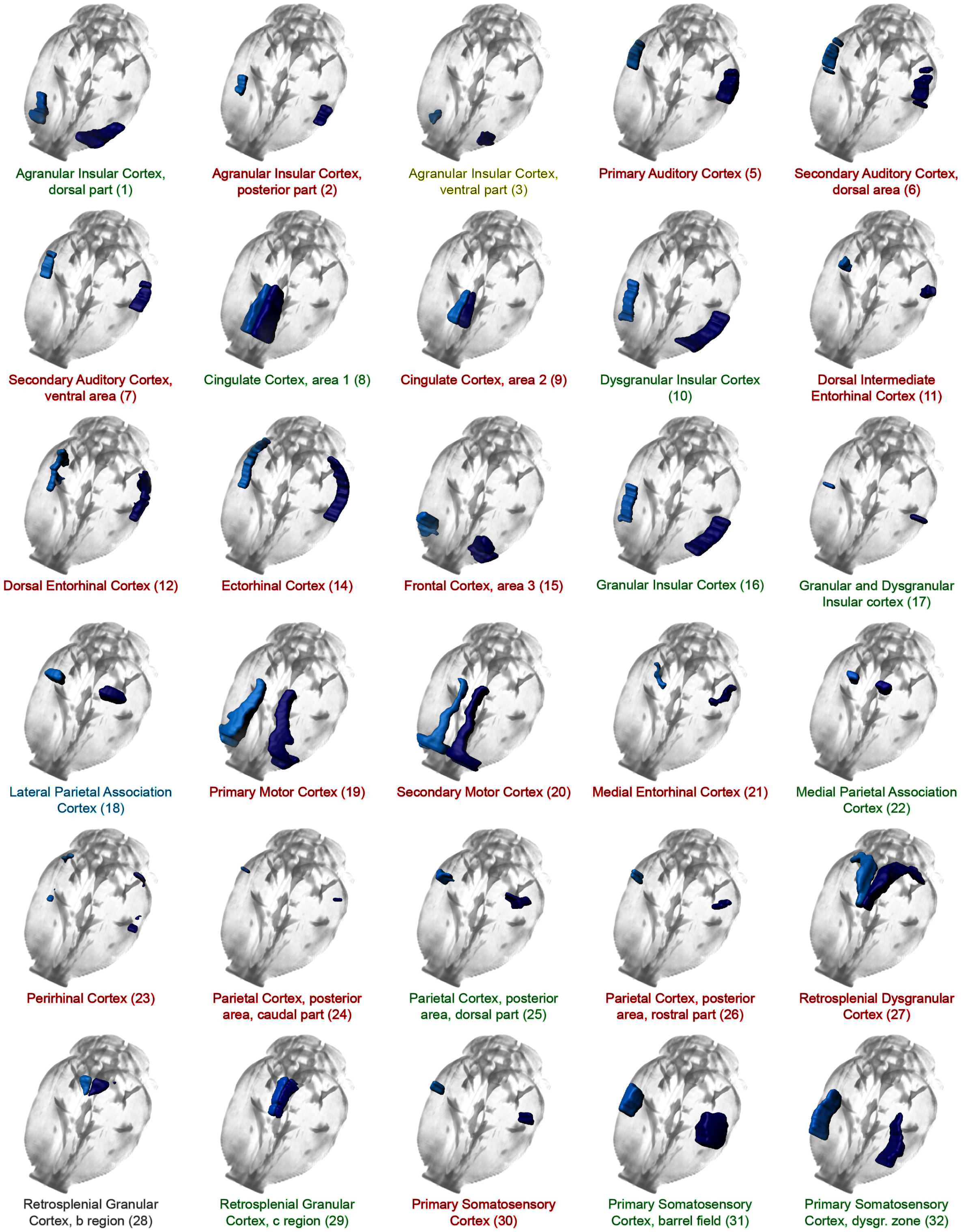
Agranular Insular Cortex, dorsal part (1)
Agranular Insular Cortex, posterior part (2)
Agranular Insular Cortex, ventral part (3)
Primary Auditory Cortex (5)
Secondary Auditory Cortex, dorsal area (6)
Secondary Auditory Cortex, ventral area (7)
Cingulate Cortex, area 1 (8)
Cingulate Cortex, area 2 (9)
Dysgranular Insular Cortex (10)
Dorsal Intermediate Entorhinal Cortex (11)
Dorsal Entorhinal Cortex (12)
Ectorhinal Cortex (14)
Frontal Cortex, area 3 (15)
Granular Insular Cortex (16)
Granular and Dysgranular Insular cortex (17)
Lateral Parietal Association Cortex (18)
Primary Motor Cortex (19)
Secondary Motor Cortex (20)
Medial Entorhinal Cortex (21)
Medial Parietal Association Cortex (22)
Perirhinal Cortex (23)
Parietal Cortex, posterior area, caudal part (24)
Parietal Cortex, posterior area, dorsal part (25)
Parietal Cortex, posterior area, rostral part (26)
Retrosplenial Dysgranular Cortex (27)
Retrosplenial Granular Cortex, b region (28)
Retrosplenial Granular Cortex, c region (29)
Primary Somatosensory Cortex (30)
Primary Somatosensory Cortex, barrel field (31)
Primary Somatosensory Cortex, dysgr. zone (32)

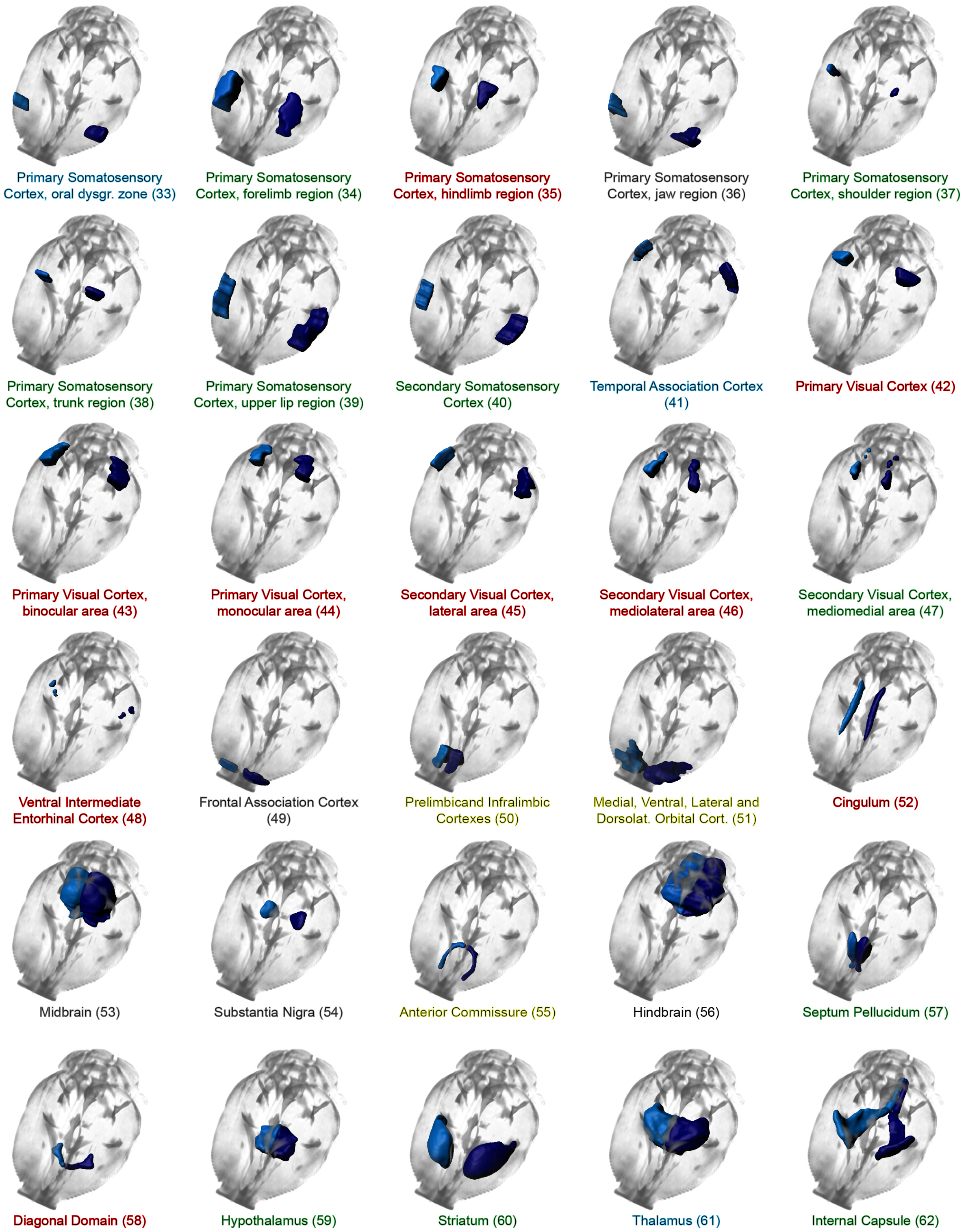
Primary Somatosensory Cortex, oral dysgr. zone (33)
Primary Somatosensory Cortex, forelimb region (34)
Primary Somatosensory Cortex, hindlimb region (35)
Primary Somatosensory Cortex, jaw region (36)
Primary Somatosensory Cortex, shoulder region (37)
Primary Somatosensory Cortex, trunk region (38)
Primary Somatosensory Cortex, upper lip region (39)
Secondary Somatosensory Cortex (40)
Temporal Association Cortex (41)
Primary Visual Cortex (42)
Primary Visual Cortex, binocular area (43)
Primary Visual Cortex, monocular area (44)
Secondary Visual Cortex, lateral area (45)
Secondary Visual Cortex, mediolateral area (46)
Secondary Visual Cortex, mediomedial area (47)
Ventral Intermediate Entorhinal Cortex (48)
Frontal Association Cortex (49)
Prelimbicand Infralimbic Cortexes (50)
Medial, Ventral, Lateral and Dorsolat. Orbital Cort. (51)
Cingulum (52)
Midbrain (53)
Substantia Nigra (54)
Anterior Commissure (55)
Hindbrain (56)
Septum Pellucidum (57)
Diagonal Domain (58)
Hypothalamus (59)
Striatum (60)
Thalamus (61)
Internal Capsule (62)

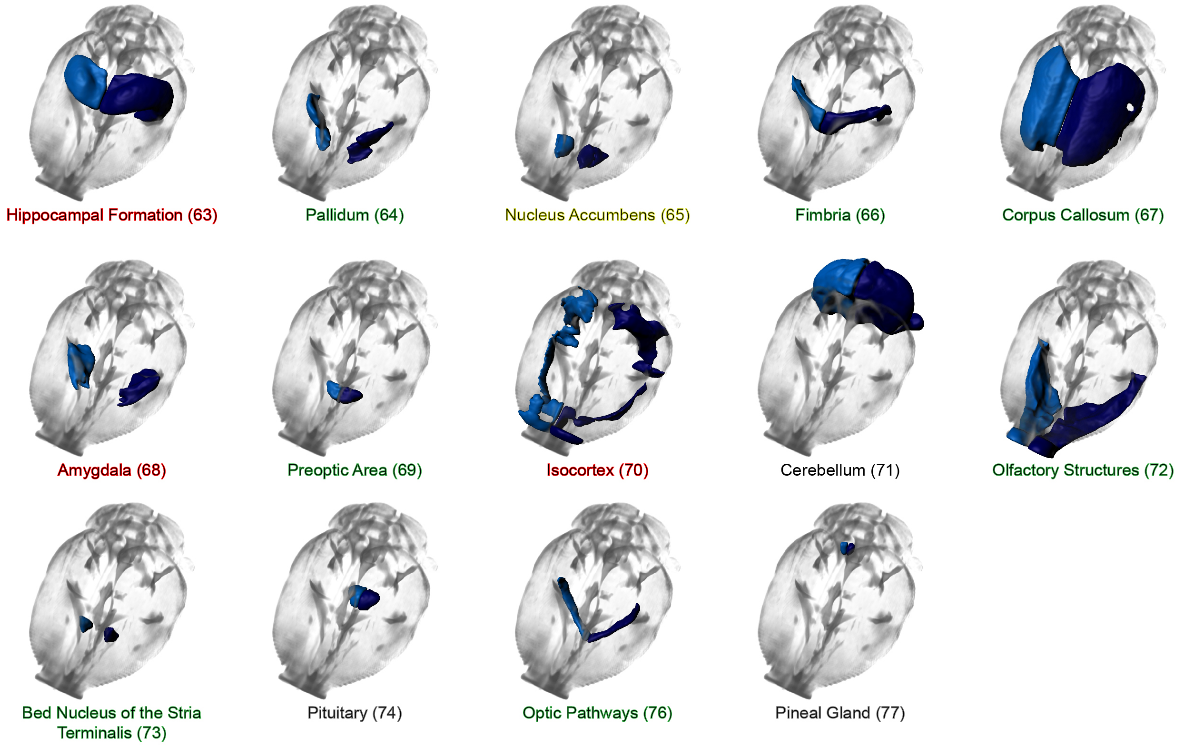

Figure S4. – 3D reconstruction of the 74 evaluated functional regions. 3D reconstruction of 74 functional rat brain regions (including cerebellum and hindbrain that were omitted from analyses due to incomplete SPECT images) based on the atlas provided by Valdes et al. 2011 (reference details can be found in the main text). Regions in the left and right hemispheres are colored dark and light blue, respectively. Area numbers based on Table S2 are shown in parentheses. Text colors correspond to the color-coding described in Figure 4B**.**

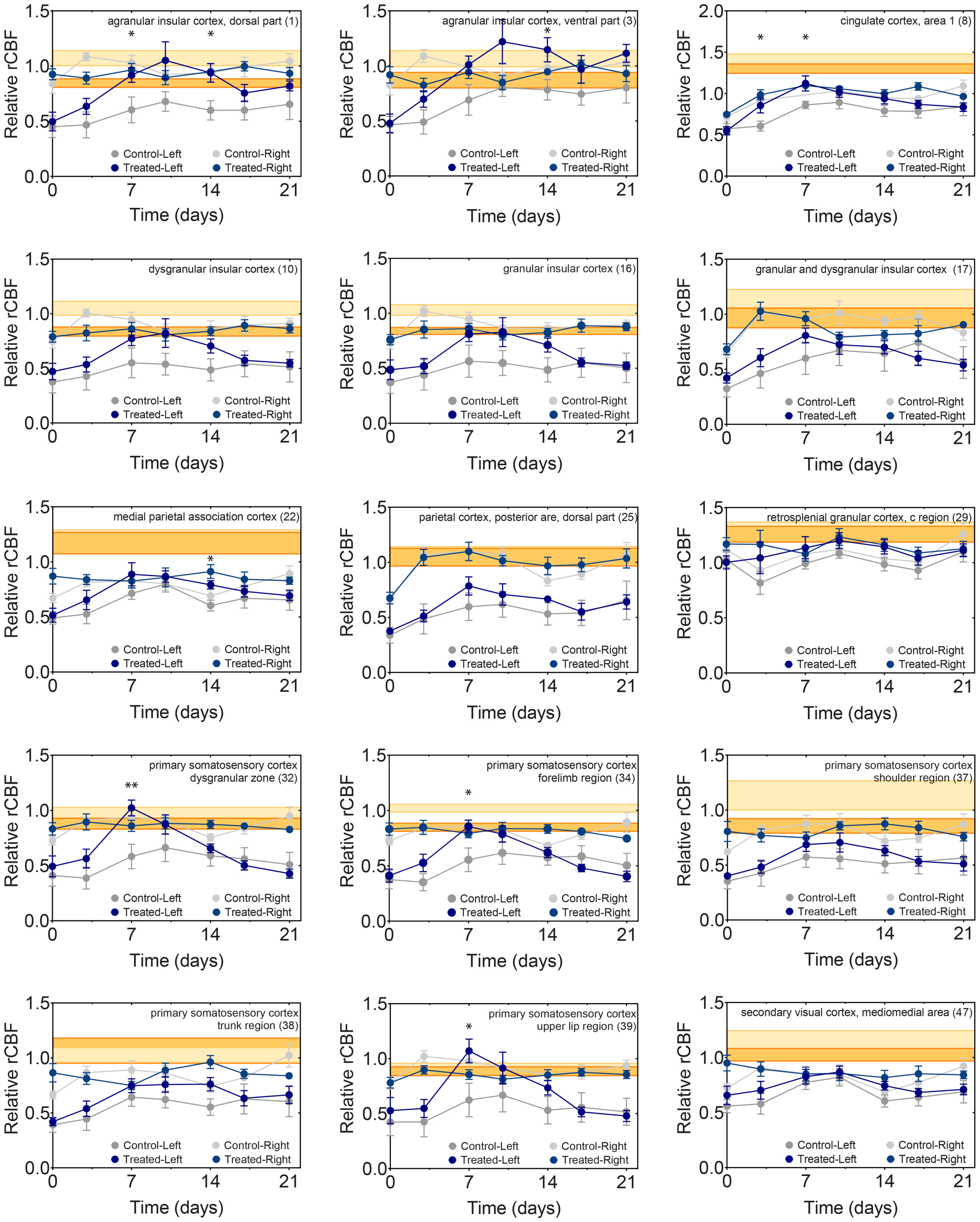
agranular insular cortex, dorsal part (1)
agranular insular cortex, ventral part (3)
cingulate cortex, area 1 (8)
dysgranular insular cortex (10)
granular insular cortex (16)
granular and dysgranular insular cortex (17)
medial parietal association cortex (22)
parietal cortex, posterior are, dorsal part (25)
retrosplenial granular cortex, c region (29)
primary somatosensory cortex dysgranular zone (32)
primary somatosensory cortex forelimb region (34)
primary somatosensory cortex shoulder region (37)
primary somatosensory cortex trunk region (38)
primary somatosensory cortex upper lip region (39)
secondary visual cortex, mediomedial area (47)
Relative rCBF
Time (days)
Control-Left
Control-Right
Treated-Left
Treated-Right

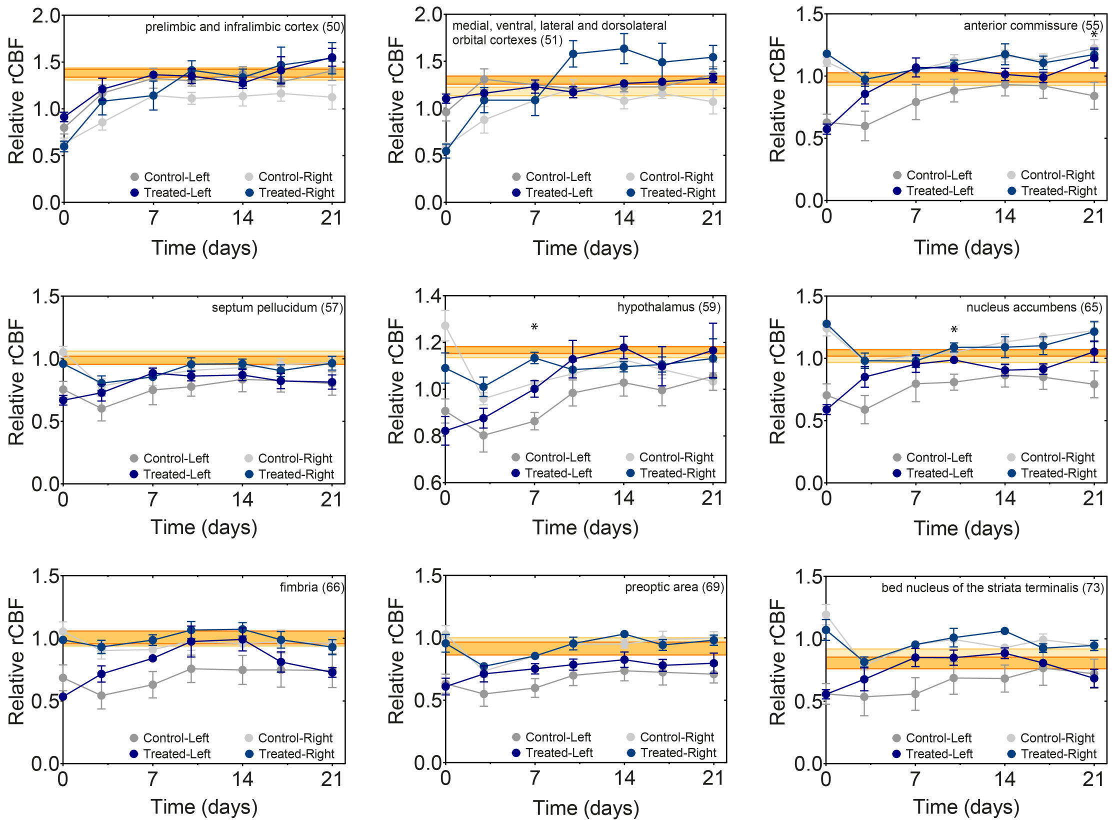

Figure S5. – Complete list of rCBF changes of functional regions. These graphs complete the list of 32 brain regions (as indicated on each panel), which showed significant improvements on day 7 after the MCAO (main Figure 4D already contains the most important 8 functional regions). Asterisks denote data points where control and treated groups differ significantly ($p < 0.05$). The bands (dark yellow – left hemisphere, bright yellow – right hemisphere) represent the mean ± SEM of physiologically normal rCBF levels calculated from the reference dataset.

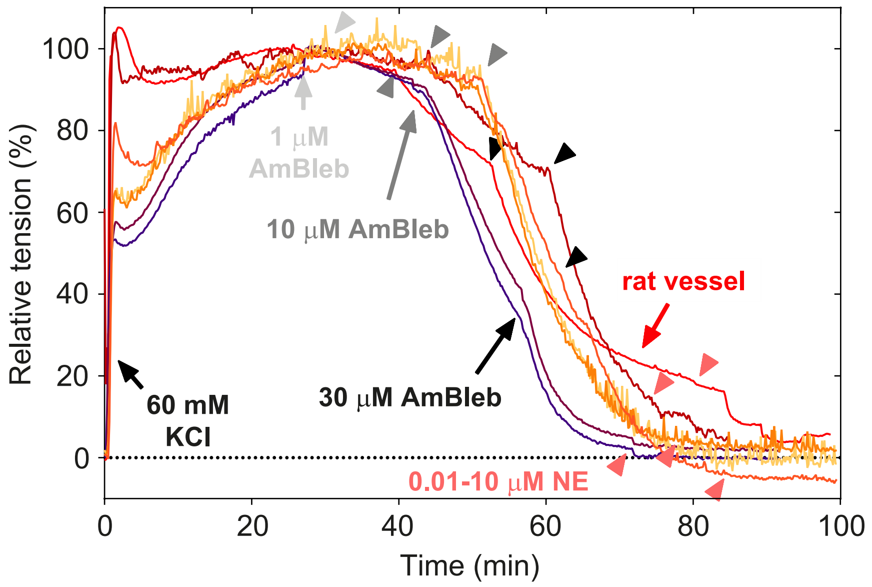

Relative tension (%)
100
80
60
40
20
0
0
20
40
60
80
100
Time (min)
1 μM AmBleb
10 μM AmBleb
30 μM AmBleb
60 mM KCl
rat vessel
0.01-10 μM NE

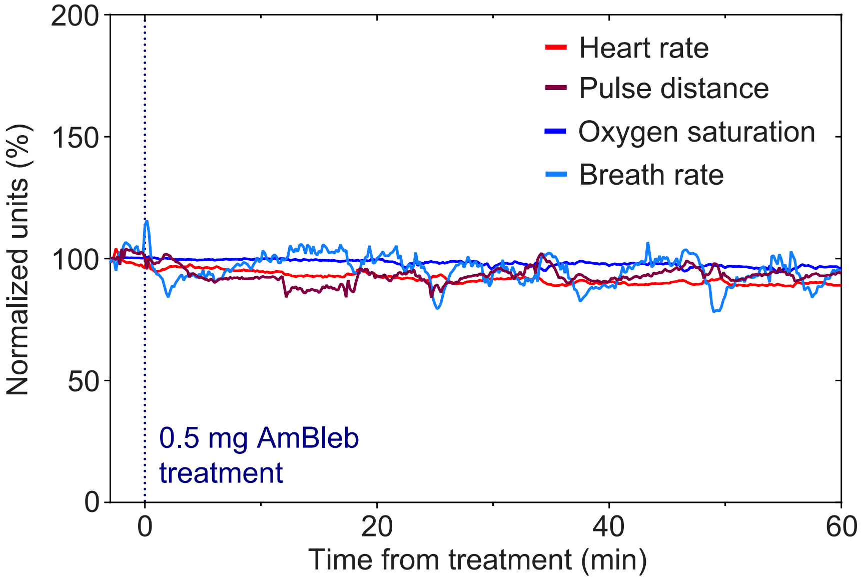
200
150
100
50
0
Normalized units (%)
0
20
40
60
Time from treatment (min)
Heart rate
Pulse distance
Oxygen saturation
Breath rate
0.5 mg AmBleb
treatment

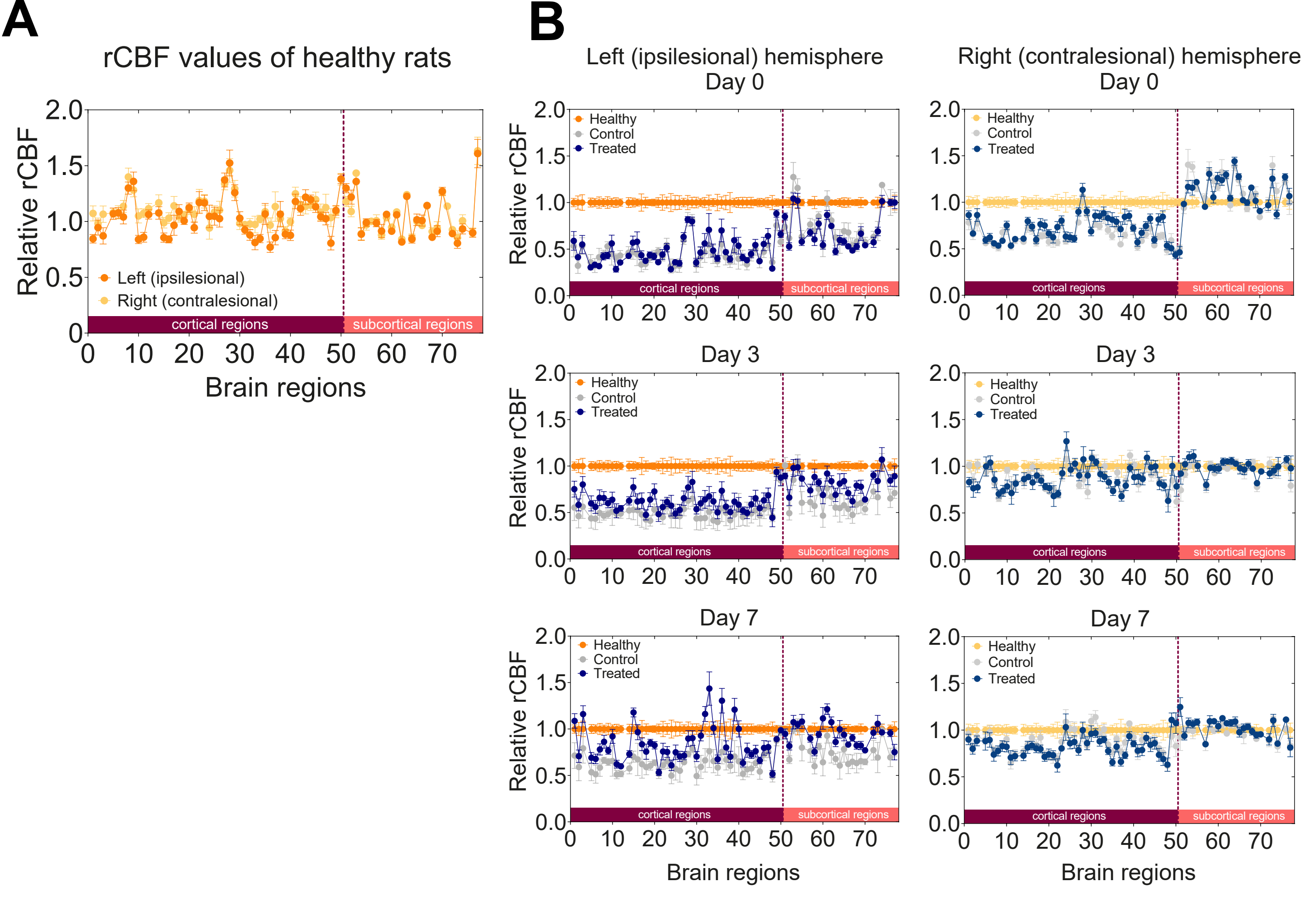
A
rCBF values of healthy rats
Relative rCBF
Left (ipsilesional)
Right (contralesional)
cortical regions
subcortical regions
Brain regions
B
Left (ipsilesional) hemisphere
Day 0
Right (contralesional) hemisphere
Day 0
Healthy
Control
Treated
Day 3
Day 7
Relative rCBF
cortical regions
subcortical regions
Brain regions

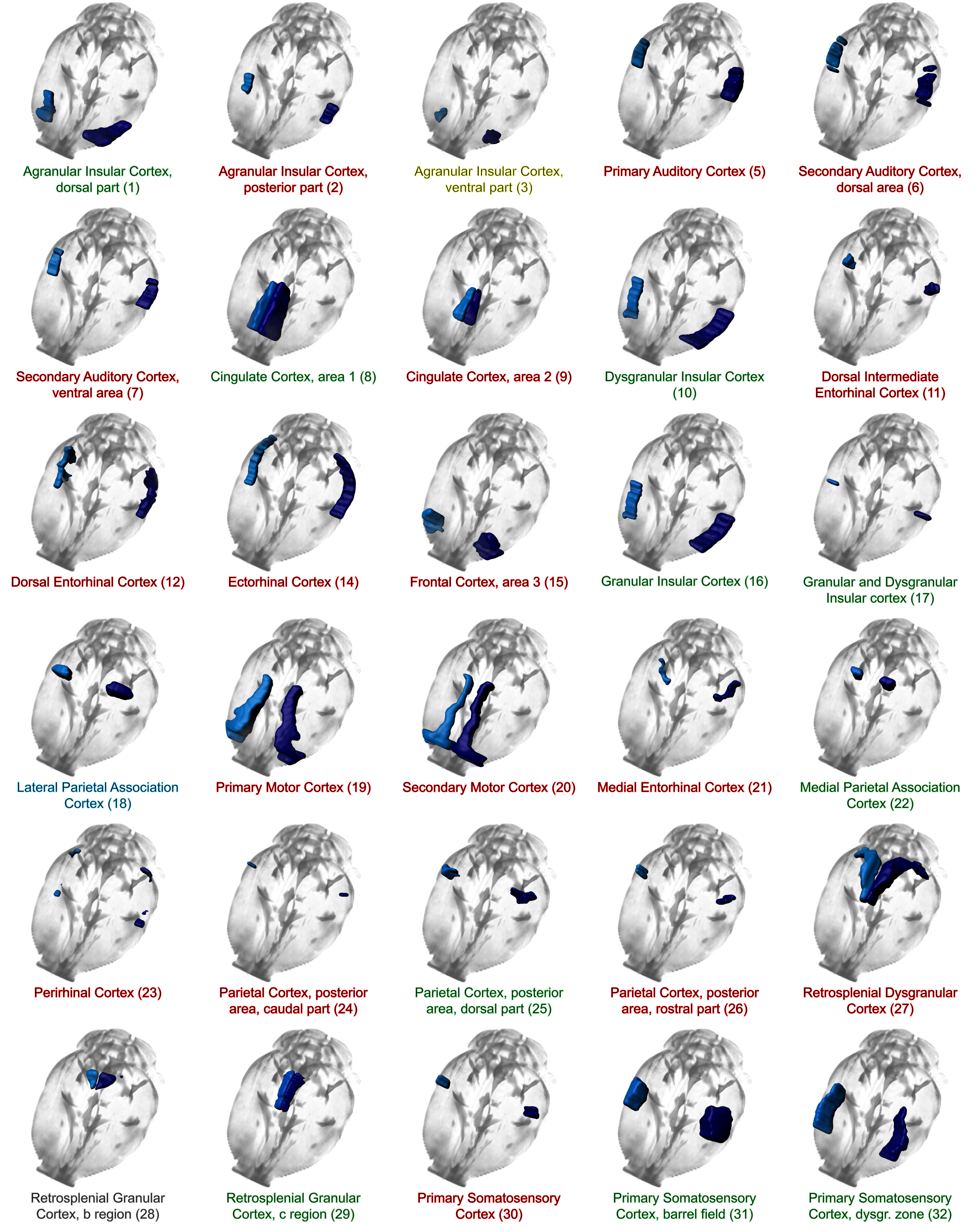

Agranular Insular Cortex, dorsal part (1)
Agranular Insular Cortex, posterior part (2)
Agranular Insular Cortex, ventral part (3)
Primary Auditory Cortex (5)
Secondary Auditory Cortex, dorsal area (6)
Secondary Auditory Cortex, ventral area (7)
Cingulate Cortex, area 1 (8)
Cingulate Cortex, area 2 (9)
Dysgranular Insular Cortex (10)
Dorsal Intermediate Entorhinal Cortex (11)
Dorsal Entorhinal Cortex (12)
Ectorhinal Cortex (14)
Frontal Cortex, area 3 (15)
Granular Insular Cortex (16)
Granular and Dysgranular Insular cortex (17)
Lateral Parietal Association Cortex (18)
Primary Motor Cortex (19)
Secondary Motor Cortex (20)
Medial Entorhinal Cortex (21)
Medial Parietal Association Cortex (22)
Perirhinal Cortex (23)
Parietal Cortex, posterior area, caudal part (24)
Parietal Cortex, posterior area, dorsal part (25)
Parietal Cortex, posterior area, rostral part (26)
Retrosplenial Dysgranular Cortex (27)
Retrosplenial Granular Cortex, b region (28)
Retrosplenial Granular Cortex, c region (29)
Primary Somatosensory Cortex (30)
Primary Somatosensory Cortex, barrel field (31)
Primary Somatosensory Cortex, dysgr. zone (32)

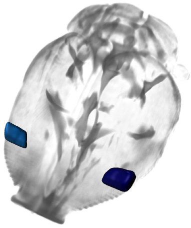
Primary Somatosensory Cortex, oral dysgr. zone (33)

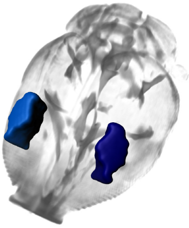
Primary Somatosensory Cortex, forelimb region (34)

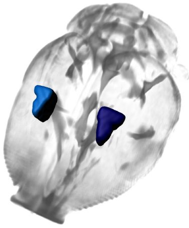
Primary Somatosensory Cortex, hindlimb region (35)

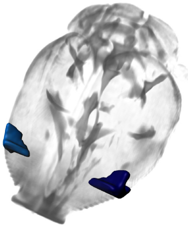
Primary Somatosensory Cortex, jaw region (36)

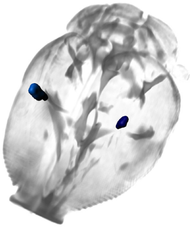
Primary Somatosensory Cortex, shoulder region (37)

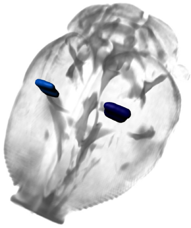
Primary Somatosensory Cortex, trunk region (38)

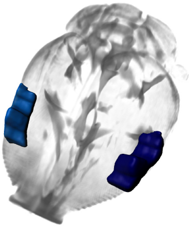
Primary Somatosensory Cortex, upper lip region (39)

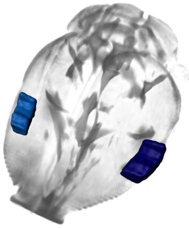
Secondary Somatosensory Cortex (40)

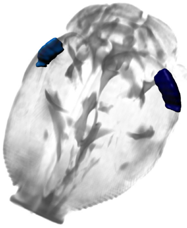
Temporal Association Cortex (41)

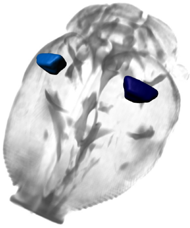
Primary Visual Cortex (42)

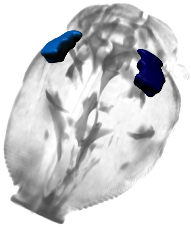
Primary Visual Cortex, binocular area (43)

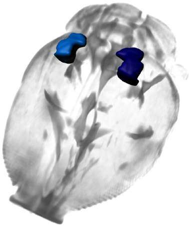
Primary Visual Cortex, monocular area (44)

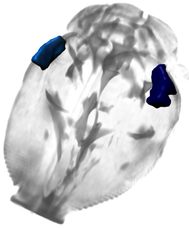
Secondary Visual Cortex, lateral area (45)

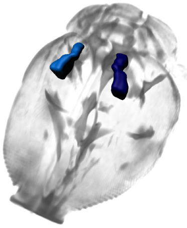
Secondary Visual Cortex, mediolateral area (46)

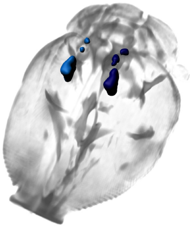
Secondary Visual Cortex, mediomedial area (47)

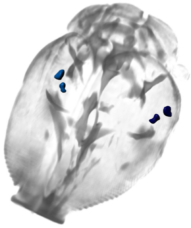
Ventral Intermediate Entorhinal Cortex (48)

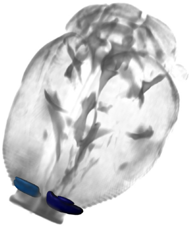
Frontal Association Cortex (49)

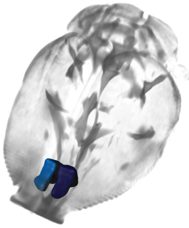
Prelimbicand Infralimbic Cortexes (50)

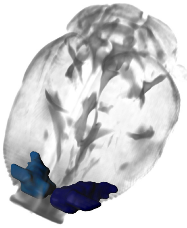
Medial, Ventral, Lateral and Dorsolat. Orbital Cort. (51)

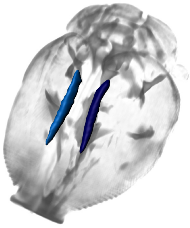
Cingulum (52)

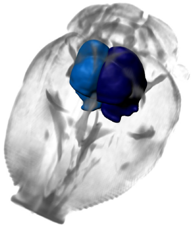
Midbrain (53)

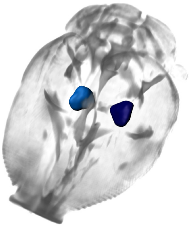
Substantia Nigra (54)

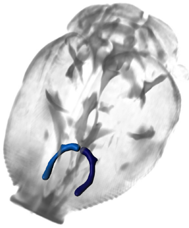
Anterior Commissure (55)

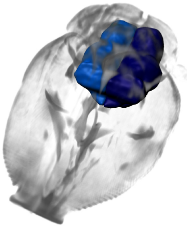
Hindbrain (56)

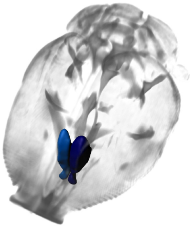
Septum Pellucidum (57)

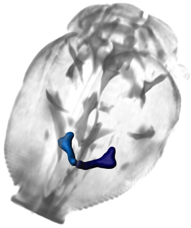
Diagonal Domain (58)

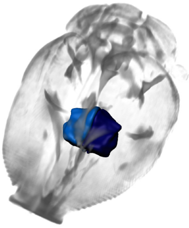
Hypothalamus (59)

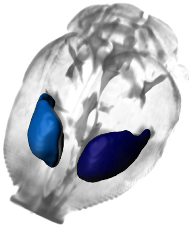
Striatum (60)

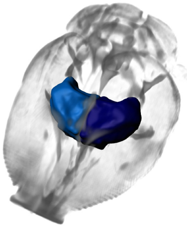
Thalamus (61)

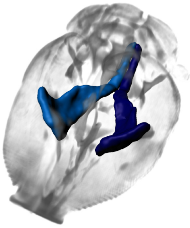
Internal Capsule (62)

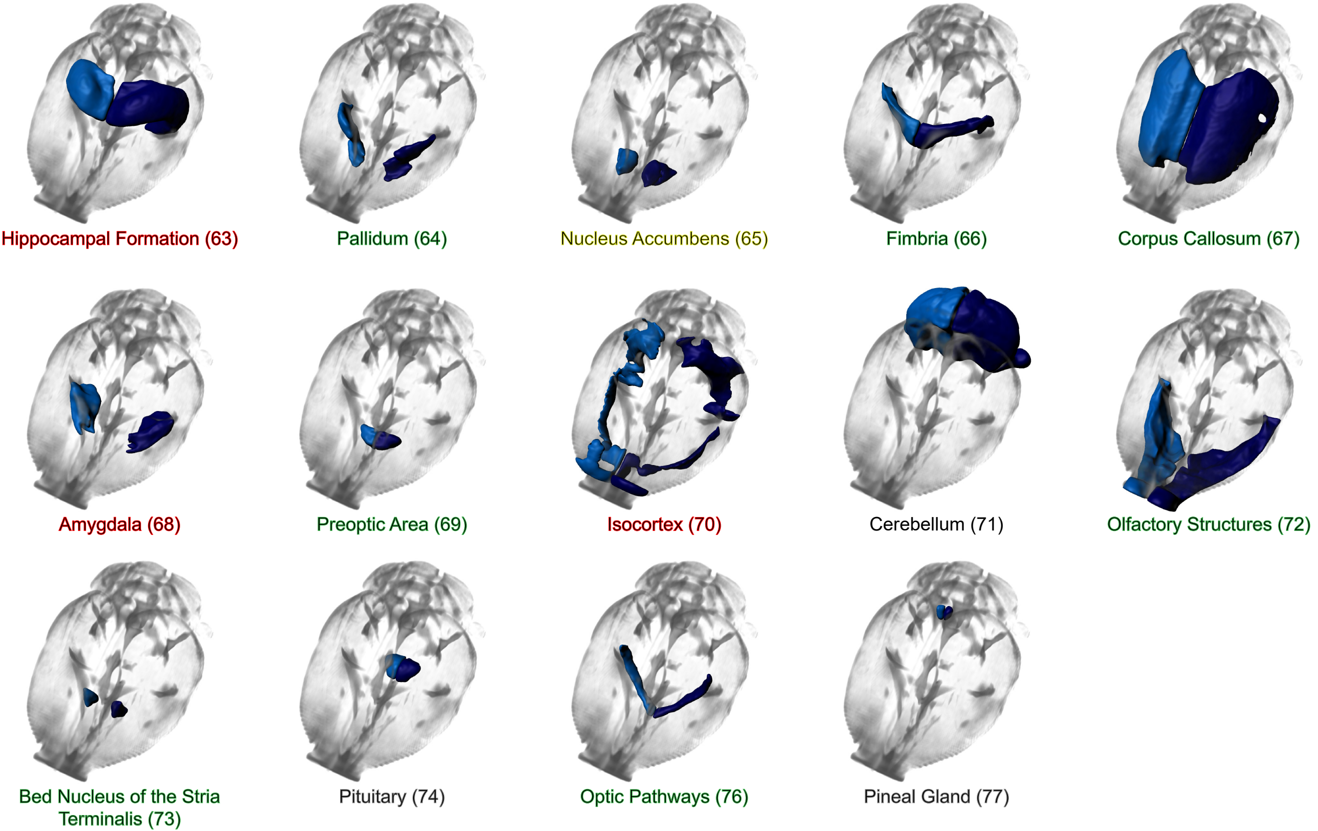
Hippocampal Formation (63)
Pallidum (64)
Nucleus Accumbens (65)
Fimbria (66)
Corpus Callosum (67)
Amygdala (68)
Preoptic Area (69)
Isocortex (70)
Cerebellum (71)
Olfactory Structures (72)
Bed Nucleus of the Stria Terminalis (73)
Pituitary (74)
Optic Pathways (76)
Pineal Gland (77)

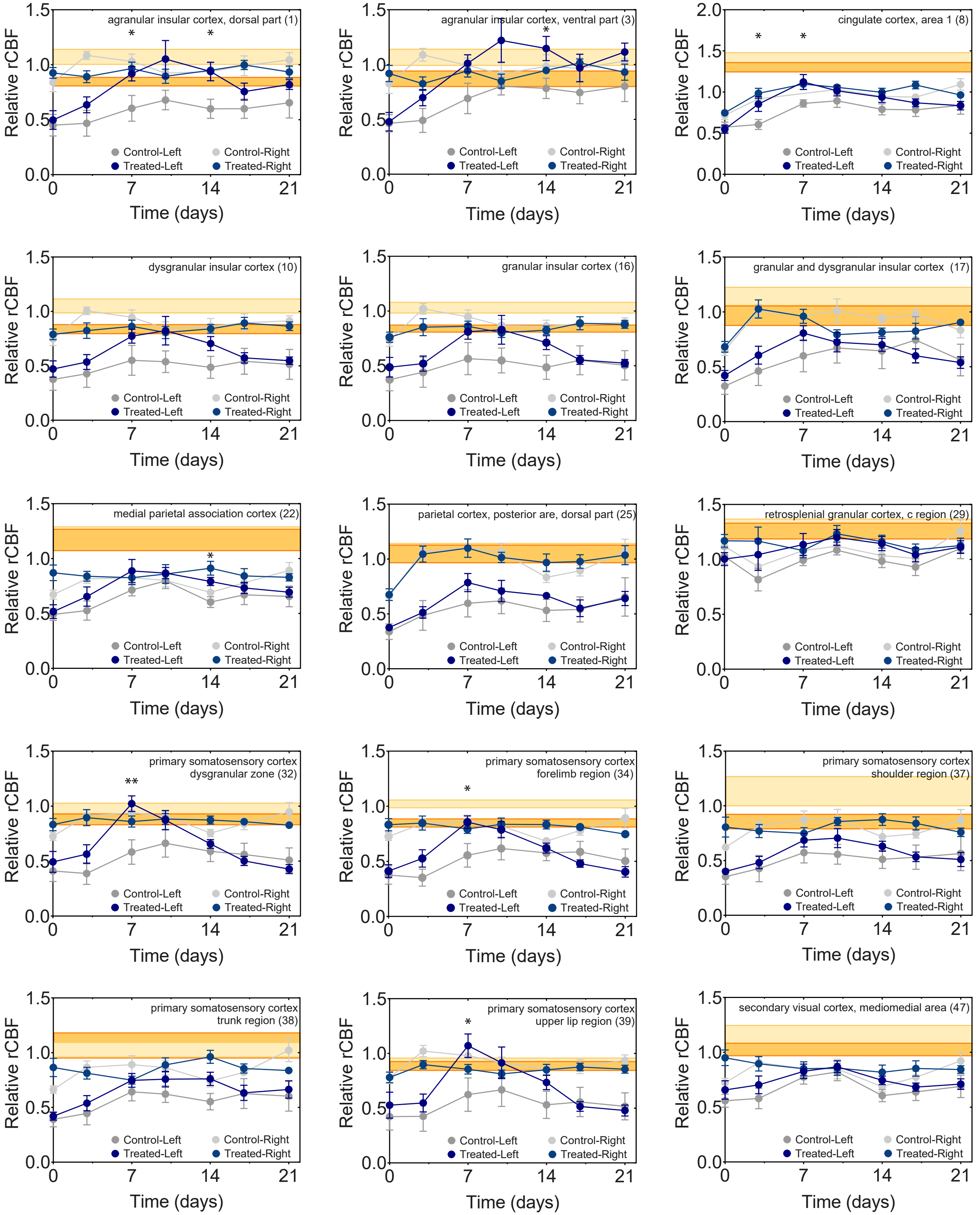
agranular insular cortex, dorsal part (1)
agranular insular cortex, ventral part (3)
cingulate cortex, area 1 (8)
dysgranular insular cortex (10)
granular insular cortex (16)
granular and dysgranular insular cortex (17)
medial parietal association cortex (22)
parietal cortex, posterior are, dorsal part (25)
retrosplenial granular cortex, c region (29)
primary somatosensory cortex dysgranular zone (32)
primary somatosensory cortex forelimb region (34)
primary somatosensory cortex shoulder region (37)
primary somatosensory cortex trunk region (38)
primary somatosensory cortex upper lip region (39)
secondary visual cortex, mediomedial area (47)
Relative rCBF
Time (days)
Control-Left
Control-Right
Treated-Left
Treated-Right

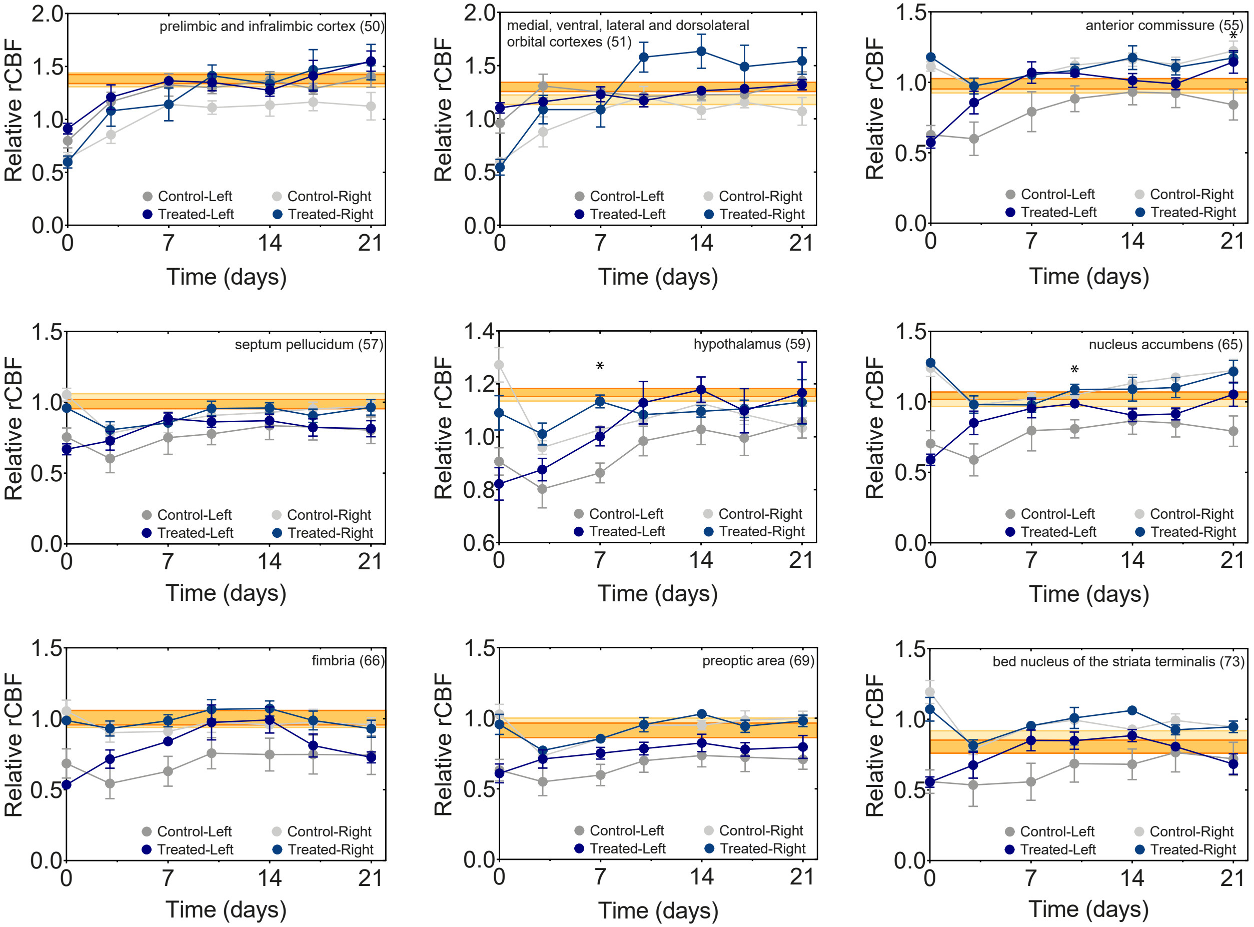

prelimbic and infralimbic cortex (50)
medial, ventral, lateral and dorsolateral orbital cortexes (51)
anterior commissure (55)
septum pellucidum (57)
hypothalamus (59)
nucleus accumbens (65)
fimbria (66)
preoptic area (69)
bed nucleus of the striata terminalis (73)
Relative rCBF
Time (days)
Control-Left
Control-Right
Treated-Left
Treated-Right
